# What Do You Think? Using Expert Opinion to Improve Predictions of Response Propensity Under a Bayesian Framework

**DOI:** 10.12758/mda.2020.05

**Published:** 2020

**Authors:** Stephanie Coffey, Brady T. West, James Wagner, Michael R. Elliott

**Affiliations:** 1 Joint Program in Survey Methodology and U.S. Census Bureau; 2 Survey Research Center, Institute for Social Research, University of Michigan-Ann Arbor; 3 Department of Biostatistics, University of Michigan-Ann Arbor

**Keywords:** Bayesian Analysis, Response Propensity, Expert Opinion, Elicitation of Priors, Responsive Survey Design

## Abstract

Responsive survey designs introduce protocol changes to survey operations based on accumulating paradata. Case-level predictions, including response propensity, can be used to tailor data collection features in pursuit of cost or quality goals. Unfortunately, predictions based only on partial data from the current round of data collection can be biased, leading to ineffective tailoring. Bayesian approaches can provide protection against this bias. Prior beliefs, which are generated from data external to the current survey implementation, contribute information that may be lacking from the partial current data. Those priors are then updated with the accumulating paradata. The elicitation of the prior beliefs, then, is an important characteristic of these approaches. While historical data for the same or a similar survey may be the most natural source for generating priors, eliciting prior beliefs from experienced survey managers may be a reasonable choice for new surveys, or when historical data are not available. Here, we fielded a questionnaire to survey managers, asking about expected attempt-level response rates for different subgroups of cases, and developed prior distributions for attempt-level response propensity model coefficients based on the mean and standard error of their responses. Then, using respondent data from a real survey, we compared the predictions of response propensity when the expert knowledge is incorporated into a prior to those based on a standard method that considers accumulating paradata only, as well as a method that incorporates historical survey data.

Responsive Survey Design (RSD; Groves and Heeringa, 2006) relies on accumulating paradata (i.e. data about the process of collecting survey data, see Couper 2000, 2017) and response data in order to introduce changes to data collection protocols or tailor data collection features to specific cases. These changes are made in pursuit of a survey goal, such as quality improvement or cost control. Unfortunately, by relying only on the partial current data as it accumulates, predictions generated from this partial data may be biased (Wagner and Hubbard, 2014) and, as a result, decisions made based on these predictions can be inefficient or even harmful.

Recently, survey researchers have introduced Bayesian approaches (Schouten et al., 2018) to mitigate this bias by supplementing the current accumulating data with prior beliefs, generated from external data such as past implementations of the same survey or the survey methodological literature (West, Wagner, Coffey and Elliott, 2019). While priors generated from past implementations of the same survey may be the most informative for a particular survey, that solution is not always an option. New surveys, or surveys whose designs have changed dramatically, may need to develop priors from different data sources. West et al. (2019) explored using a literature review to source prior information for response propensity models in the National Survey of Family Growth (NSFG). While priors from the literature review did not perform as well as priors from historical NSFG data, they outperformed model predictions made only using current accumulating paradata, particularly in the middle portion of the data collection period.

The present study evaluates another potential source of prior information. Here, expert knowledge was elicited from survey managers (“experts”), through a self-response questionnaire designed to collect their predictions of attempt-level response rates, or changes in those expected response rates, for various types of sample members. Given those survey responses, pooled priors were created from expert respondent data. The structure of the items in the questionnaire completed by the experts mimicked that of the existing response propensity model. We then evaluated these priors’ ability to improve predictions of response propensity in the National Survey of Family Growth (NSFG) relative to only using partial data from the current round or using historical data as an alternative source for the development of priors. This manuscript discusses the content of the questionnaire, the identification of experts, the method for generating priors, and an evaluation of how the information from expert elicitation affects the bias and root mean squared error (RMSE) of the daily predictions of response propensity. We found that priors based on expert opinion led to modest improvements in prediction during the middle and late portions of data collection when compared to using only current round data. Additionally, we found that priors based on expert opinion were sometimes competitive with, though generally did not outperform, an approach that used historical data evaluated in West et al. (2019). We also identified several ways to improve upon our elicitation process that may lead to further improvements in predictions based on expert opinion over methods more commonly used in RSDs.

## Background

### Responsive Survey Design

Responsive survey design (RSD; Groves and Heeringa, 2006) has emerged as a framework for maintaining or improving survey outcomes in an increasingly difficult survey climate. Increasing data collection costs, and decreasing cooperation and response rates, have caused survey methodologists and managers to explore alternatives to the prevailing “one path fits all sample members” approach to data collection operations (Axinn, Link and Groves, 2011). Instead, RSD uses accumulating paradata and response data to make changes to later data collection protocols. These changes attempt to increase data quality in some specified way or control costs, relative to continuing with the standard data collection protocol. Types of protocol changes may include introducing another mode (Coffey, Reist and Miller, 2019), changing the effort spent on specific cases (Rosen et al., 2014), or a change in tokens of appreciation combined with subsampling (Wagner et al., 2012).

In an RSD, one of the most common ways to tailor data collection features to specific cases is with predicted propensity scores. Based on frame data and accumulated paradata, these predictions can be used to alter data collection operations. Various surveys have utilized propensity scores to differentially implement a variety of data collection features, including protocol assignment (Peytchev, Rosen, Riley, Murphy and Lindblad, 2010; Roberts, Vandenplas and Stahli, 2014), incentives (Chapman, 2014), and allocation to nonresponse follow-up (Laflamme and Karaganis, 2010; Thompson and Kaputa, 2017) in hopes of improving survey outcomes.

Paradata from the current round of data collection provide useful predictors of survey outcomes, such as response propensity, for the sampled cases currently receiving recruitment effort. In an RSD, targeted interventions are applied to cases during the data collection period in order to shift response propensities in pursuit of a cost- or quality-related survey goal, necessitating high quality predictions of these propensities. However, during the survey period when an RSD would be implemented, the accumulating paradata are “incomplete” relative to the final data, in that completed cases and incoming data from early in the data collection period may not be representative of that which will be collected later in data collection. As a result, only using the accumulating data from the current round of data collection could result in biased predictions of response propensity (Wagner and Hubbard, 2014) or reduced prediction performance when predicted propensities are classified into response categories, either of which could lead to inefficient decisions. In this paper, we focus on the error in the predictions of response propensity scores, as opposed to the secondary step of classification error.

In order to improve predictions, survey practitioners often use external data that may be more representative of a full data collection period. It is relatively common to estimate the coefficients of a predictive model using historical data, such as a prior implementation of the survey, and then apply those coefficients to the current round of data collection (Schouten, Calinescu and Luiten 2013; Schouten, Wagner and Peytchev, 2017; Schouten, Mushkudiani, Shlomo, Durrant, Lundquist and Wagner, 2018). While this method provides data that might be representative of an entire data collection, it ignores current data in the prediction process.

More recently, survey researchers have begun exploring Bayesian approaches that utilize both external and current data in the prediction process. Prior beliefs are generated from external data, most commonly historical data from the same survey, and those priors are then updated as the current data accumulates. Schouten et al. (2018) discuss using Bayesian methods for predicting response and cost under different scenarios. Through simulation, they demonstrate value in the Bayesian methods in terms of reduced RMSE of predictions, while stressing that misspecification of the priors with respect to the true data should be relatively small. Empirical evidence is also emerging (West et al., 2019) that combining published estimates or historical information and current round information in a Bayesian setting can improve prediction.

### Empirical Evidence and Sources of Prior Information

West et al. (2019) compared the performance of predictions of response propensity in the NSFG, a nationally representative quarterly survey in the U.S., when Bayesian methods are used versus when only current data is used. The Bayesian methods incorporated external information in the form of priors, either from past implementations of the NSFG or from published research on propensity models found through a literature review. Results demonstrated that the Bayesian approaches consistently reduced both the bias and the mean squared error (MSE) of predicted response propensities, particularly in the middle of data collection, when an RSD may be implemented. This was true for either source of prior information -- the historical data or the literature review.

The quality of the prior information is directly related to its ability to improve predictions of interest, and so the source of prior information is an important consideration. It seems reasonable that historical data from the same survey would result in the most informative priors for the prediction of interest; however, there may be cases where this information is not available. New surveys, for example, would not have access to historical information. Additionally, surveys that have undergone significant redesign, such as introducing a new mode, changing an incentive amount, or dropping a screening interview, may find that priors based on historical paradata are no longer available.

There may be cases where even a literature review produces limited or no useful external information. In the case where a survey has an unusual or unique target population, or the prediction of interest is not as common as response propensity, there may not be sufficient information in the literature from which to develop priors. In these cases, where there is an absence of objective information, expert opinion may be the only option for generating the necessary information for prior construction. Expert opinion is often used implicitly in survey planning – experienced survey managers may provide input into expected response rates to help determine sample sizes, or for estimating budgets. Additionally, they may help explain variation in progress or response rates during data collection. Transforming expert opinion into priors explicitly incorporates this information into the prediction model.

### Expert Elicitation

Clinical trials and health care evaluations often rely on prior beliefs for a variety of reasons. Dallow, Best and Montague (2018) describe a protocol for eliciting expert opinion in order to improve the drug development process. Mason et al. (2017) propose a practice for leveraging expert opinion in the analysis of randomized controlled trials when there are missing observations for patients. Additionally, Boulet et al. (2019) demonstrate the use of expert opinion in a variable selection process for personalized medicine. When novel treatments are tested, or prior trials have very small sample sizes or are otherwise not comparable, expert opinion can be relied upon for developing priors (Hampson, Whitehead, Eleftheriou and Brogan, 2014).

Spiegelhalter et al. (2004, Ch. 5) as well as O’Hagan (2019) provide overviews of the expert elicitation process, and the potential biases that may arise in priors elicited from individuals. *Availability bias* may arise when experts are asked about easily recalled events – they may estimate a higher or lower probability than is accurate. For example, if survey experts have recently seen frequent reports of language barriers along with increasing non-interview rates, the experts may inflate the effect that a language barrier has on overall response rate or response propensity, even if there are other contributing factors to increasing non-interview rates. *Anchoring bias* may lead experts to shrink intervals between different categories or groups based on a provided piece of information or their initial elicited quantity or probability. Once an expert learns from the elicitation instrument, or offers through the elicitation process, that the expected response rate for one group is 45%, future answers about different subgroups may be biased towards 45%.

*Overconfidence bias* may lead to distributions of the priors with insufficient variance. This may occur when elicitation happens in small groups and some strongly opinionated experts convince others of their opinion, a behavior also known as groupthink. Alternatively, in individual elicitation, overconfidence bias may arise because of the expectation of experts that they have, in fact, a greater amount of expertise than they actually do, resulting in under-reported uncertainty. *Conjunction fallacy bias* may arise when a particular event is given a higher estimated probability when it is the subset of another event. For example, on any given contact attempt, the probability that any open case will have had a callback request and respond is necessarily smaller than the probability that any open case will respond. However, an expert may suggest the opposite, thinking that having a callback request makes response much more likely. This bias is often due to the rarity of one of the two events, which in this case would be the callback request. Finally, *hindsight bias* may arise if the expert is asked to provide a prior expectation after looking at the current data. Awareness of all of these types of bias is useful in the design of the expert elicitation process.

Spiegelhalter et al. (2004, Ch. 5) also discuss four common methods for elicitation: informal discussion, structured interviewing, structured questionnaires, and computer-based elicitation. Each of these methods requires different amounts of interaction with experts, and allows for different levels of complexity of prior development. Additionally, these authors discuss three methods for combining information when multiple experts are utilized: arriving at a consensus value among all experts, arithmetic pooling, or retaining individual priors. O’Hagan (2019), whose elicitation method elicits distributions from experts, discusses the combination of those distributions to generate a pooled empirical distribution for the prior.

Here, we adapted the concept of expert elicitation of priors from the clinical trials literature. Our goal was to evaluate whether expert opinion can be helpful when little objective data is available for generating priors for the coefficients in a logistic regression model used to estimate propensity of response. In this application, we elicited opinion from experts independently through an internet questionnaire, and used arithmetic pooling to combine the elicited information into priors for models used to generate daily predictions of response propensity in the NSFG.

## Data and Methods

### Overview of the National Survey of Family Growth

The NSFG is conducted by the National Center for Health Statistics, under contract with the Institute for Social Research (ISR) at the University of Michigan. The NSFG, in its current iteration, is a cross-sectional survey for which data were collected continuously throughout the calendar year from 2011–2019. In a given year, four data collection operations are conducted, with data being collected from four independent, nationally representative samples. The field operations for each sample last three months, or one quarter (e.g., January to March, April to June). The survey selects a national sample of U.S. housing unit addresses each quarter of the year. The target population from which the NSFG selects these four independent national samples is 15 – 49 year old persons living in the U.S. (Lepkowski, Mosher, Groves, West, Wagner and Gu, 2013). The NSFG is a two-stage survey, meaning there is first a screener interview to determine eligibility, followed by the main interview. Interviewers first visit randomly sampled households and attempt to screen the households for eligibility. Within eligible households, one of the eligible individuals is randomly selected to complete the main survey interview, which usually takes 60–80 minutes and covers a variety of fertility-related topics.

NSFG paradata are aggregated on a daily basis and used to predict the probability that active households will respond to either the screening interview or the main interview. Survey managers might use these predictions for prioritization of active cases (e.g., Wagner et al., 2012) or for stratifying the sample when selecting a subsample of active cases for the new data collection protocol after 10 weeks (Wagner et al., 2017). At this point, managers may oversample high-propensity cases, or offer a higher token of appreciation to encourage response. Accurate model-based predictions are thus essential for maximizing the efficiency of the data collection effort in any given quarter. For purposes of this study, we focus on models for the probability of responding to the initial screening interview.

### Response Propensity Models in the NSFG

For this application, we used data from five quarters of the NSFG (Quarters 16 – 20), covering the June 2015 to September 2016 time period. For each of the five quarters, our prediction of interest was the probability of response to the screening interview at the next contact attempt, using either the current accumulating paradata only, or the combination of priors generated from expert elicitation and the current accumulating paradata. We also compared these methods to the best performing method in West et al. (2019), which combined current accumulating paradata with priors based on historical data from the eight preceding quarters of data collection.

In order to compare predictions generated from our proposed method with those discussed in West et al (2019), we used the same predictive modeling approach (discrete time logistic regression), and the same set of predictors of screener response propensity. In that paper, eight quarters (or two years) of the NSFG (Quarters 13 – 20) were combined into a stacked dataset containing all contact attempt records and a binary outcome for each record that indicated whether the screener interview was completed on that particular attempt or not. The authors then fit a discrete time-to-event logistic regression model to this dataset to identify significant predictors. Available predictors included sampling frame information, linked commercially-available data, and NSFG paradata, all of which have been used to predict response propensity in the NSFG (West, 2013; West and Groves, 2013; West et al., 2015). The authors used a backward selection approach to model-building, retaining all predictor variables that appeared in all eight quarters with a p-value less than 0.05 based on a Wald test for all regression parameters associated with a given variable.

They then included two predictor variables that were important for sampling and weighting in order to control for sampling domain in the response propensity model. The first was the sociodemographic domain of each housing unit, based on the percentage of the population in the Census Block Group containing the segment that is Black and/or Hispanic as reported in U.S. Census data. The second was a three-level categorical variable indicating whether a case was in a self-representing area, a non-self-representing metropolitan statistical area (MSA), or a non-MSA non-self-representing area. Self-representing sampling areas are geographic sampling domains that are large enough to be sampled with certainty in a probability proportionate-to-size sample, and, therefore, represent only themselves during weighting and estimation. These two variables were initially included in the backwards selection procedure, but were not found to be statistically significant, and so were not retained. However, after consultation with data collection managers, these two variables were added back into the response propensity model in order to control for sampling domain in the predictive model.

All retained predictors from the backward selection process carried out in West et al. (2019), including their estimated coefficients and standard errors, are listed in table A1 in the online appendix. Several predictors came from each available data source: the sampling frame, commercially-available data, and paradata. By using the same list of predictors, and the same discrete-time logistic regression model specification, we are able to compare the effect that priors based on expert elicitation have on the predictions of response propensity, versus excluding prior information, or using priors from historical data. The focus of our analysis is on the relative performance of these methods given a particular model.

### Design of Prior Elicitation Process

For this proof-of-concept study, we wanted our prior information to be based upon a relatively large group of experts to generate a reasonable distribution from which to estimate priors. Our target sample size meant that elicitation methods requiring significant interaction with experts, including informal discussion and structured interviewing, were not feasible. As a result, we created and distributed a structured questionnaire to selected experts, who could then respond at their convenience. The questionnaire asked experts to provide their opinions on attempt-level response rates for subgroups with various types of characteristics, and, in some cases, opinions on changes to response rates based on certain characteristics.

The questionnaire included the significant predictors found in the retrospective analysis of the NSFG response propensity model, as described in Section 3.2. These predictors include items from the sampling frame, including geographic and sampling strata information, as well as time-varying attempt-level information, derived from accumulating paradata. Fixed characteristics include sampling frame or commercially available data, like the 9-level Census Division geographic variable. In the questionnaire, we asked experts their opinions on their expected response rates for each of the nine categories. Time-varying covariates were based on paradata and include indicators for past contact or instances of the sample member expressing questions, comments or concerns. In the questionnaire, we requested information about the expected change in response rate for characteristics like each additional contact attempt, or whether the sample member expressed comments on concerns on the most recent contact attempt. We also asked experts to provide their experience with survey data collection by selecting one of three categories: 0 to 4 years, 5 to 15 years, and 15 or more years.

We solicited feedback from two survey experts prior to distributing the questionnaire in order to get basic feedback about content, complexity, and readability. In some cases, edits resulting from this initial feedback changed the format of the questions to make them easier to understand and answer. This meant that the format of the questions did not always match the format of the predictor in the propensity model. The final version of the questionnaire can be found in the online appendix, and in the Center for Open Science repository (https://osf.io/3kxzb) at the Open Science Framework (log-in required).

Given the target number of experts, we opted to develop priors through arithmetic pooling of all respondent information. At the same time, we wanted to avoid the biases mentioned by Spiegelhalter et al. (2004, Ch. 5). In order to avoid anchoring bias while still eliciting reasonable responses, we provided an overall expected attempt-level response rate (24%), but did not provide anchor points for any particular category in the survey, allowing the experts to provide input for all items and categories. To avoid hindsight bias (Schouten et al., 2018) arising from the fact that experts at ISR also conduct the NSFG, we recruited additional experts from the U.S. Census Bureau (Census). These additional experts have experience managing interviewer-administered data collections, but do not have experience with the NSFG or its data. By soliciting predictions from two geographically dispersed survey organizations with varying familiarity with the NSFG, we also hoped to protect against overconfidence bias (Schouten et al., 2018), which can lead to prior distributions that are too narrow and do not accurately reflect the uncertainty in the prior.

At both ISR and Census, we worked with senior survey managers to identify experienced interviewer supervisors, field directors, and survey methodologists who were knowledgeable about survey processes and reviewed progress data on a daily basis as part of their job responsibilities. We recruited eight individuals from ISR, and 12 from Census (two from each of the six regional offices). During March 2019, the recruited experts were asked to complete the questionnaire, and were encouraged to provide feedback, either directly or through a scheduled debriefing. We summarize the feedback received in the Results section.

### Method for Deriving Priors

We obtained 20 sets of expert responses about the effects on attempt-level response rates of various characteristics of sample members and paradata items, subject to some item nonresponse. We used arithmetic pooling to combine the priors and generate an expected mean and standard error for a coefficient in an attempt-level response propensity model (Spiegelhalter et al., 2004, Ch. 5).

Before pooling, however, we had to convert the estimates of differences in response rates to model coefficients for use in a logistic regression model. When categorical variables are included as predictors in a logistic regression model, the estimated coefficients are generally interpreted with respect to a reference category. Therefore, the mathematical manipulation involved identifying a reference category, calculating odds ratios with respect to the reference category, and then taking the natural log of the odds ratio to obtain a logistic regression model coefficient, or beta. We first did this for each respondent’s information individually.

Formula 1 below demonstrates how to calculate the coefficient for the *k*^*th*^ category of the *j*^*th*^ item for the*i*^*th*^ expert, ${\hat{\beta }}_{ijk}$, given the estimated probability of response for category *k* of interest, ${\hat{p}}_{ijk}$, and the estimated probability of response for a reference category *R*, ${\hat{p}}_{ijR}$.

(1) $${\hat{\beta }}_{ijk}=ln\left(\frac{{\hat{p}}_{ijk}/\left(1-{\hat{p}}_{ijk}\right)}{{\hat{p}}_{ijR}/\left(1-{\hat{p}}_{ijR}\right)}\right)$$

Using gender as an example (abbreviated *G* in the expression below), assume that the *i*^*th*^ respondent estimates the expected call-level response rate for female sample members to be 85% (as opposed to 70% for males), and male is the reference category. The *beta* for female sample members, for the *i*^*th*^ expert, would be:

$${\hat{\beta }}_{iGF}=ln\left(\frac{{\hat{p}}_{iGF}/\left(1-{\hat{p}}_{iGF}\right)}{{\hat{p}}_{iGM}/\left(1-{\hat{p}}_{iGM}\right)}\right)=ln\left(\frac{0.85/(1-0.85)}{0.70/(1-0.70)}\right)=0.8873$$

Continuous variables were converted to model parameters using the same formula but with a slightly different explanation. For these items in the questionnaire, expert opinion was elicited about the *change* in response propensity, given some unit change in the continuous variable. For example, survey managers were asked to provide their expected change in response rate for each additional contact attempt made on a sample member, and a survey manager might have responded saying they would expect a −10% change, or a 10% reduction, in response propensity for each additional contact attempt.

However, unlike standard linear regression, where there is linear change for every unit increase, logistic regression results in exponential change for each unit increase, meaning the change in response propensity is dependent on *which* unit increase is being considered (e.g. from 1 to 2 attempts, or from 8 to 9 attempts). In the case of continuous variables, we did not have a defined reference category, and so the reference is always to the average attempt-level response rate of 24%.

If the *i*^*th*^ expert believes that increasing the number of contact attempts, *j*, by one would change the attempt-level response rate by some amount, we can adapt Equation (1) above for a continuous variable. While we do not have a defined reference category, we have the overall average attempt-level response rate, 24% and the expected change provided by the expert, 5%. This results in a model coefficient of:

$${\hat{\beta }}_{ij}=\ln\left(\frac{\;\text{odds}\;(\;\text{attempts}\;=(n+1))}{\;\text{odds}\;(\;\text{attempts}\;=(n))}\right)=ln\left(\frac{0.29/0.71}{0.24/0.76}\right)=0.2573\;.\;$$

We note at this point that, while we have elicited priors on a linear scale, linking these back to the logistic scale changes the interpretation. We provide more consideration of this issue in the Discussion section.

To pool the expert information, we then took an arithmetic mean, ${\hat{\bar{\beta }}}_{jk}$ (or ${\hat{\bar{\beta }}}_{j}$ for continuous items), of the coefficients from the expert respondents. The standard error of the prior, $SE\left({\hat{\bar{\beta }}}_{jk}\right)$, was estimated by dividing the standard deviation of the coefficients from the respondents by the square root of the number of respondents, *n*.

(2) $${\hat{\bar{\beta }}}_{jk}=\frac{1}{n}\sum_{i=1}^{n}{\hat{\beta }}_{ijk}$$

(3) $$SE\left({\hat{\bar{\beta }}}_{jk}\right)=\sqrt{\frac{1}{n(n-1)}\sum_{i=1}^{n}{\left({\hat{\beta }}_{ijk}-{\hat{\bar{\beta }}}_{jk}\right)}^{2}}$$

We chose to transform each expert response into an odds ratio, take the log, and then pool the individual log-odds ratios for a few reasons. Mathematically, by first transforming each expert response into a log-odds ratio before pooling, we are working under the assumption that the log-odds are normally distributed, as opposed to the response rate or response propensity, which is how the experts provided their opinions. We felt this assumption was reasonable. First, response rates and response propensities are bounded at (0,1), and are not normally distributed, whereas the log-odds can take on any number on the real line. Additionally, the log-odds is a linear function, while the function for the odds (and for probabilities) are multiplicative and exponential, which suggests that the log-odds might converge to a normal distribution more quickly than the odds, given enough sample size.

Operationally, by generating a model coefficient for each expert, we were able to calculate a mean and standard error for each model coefficient. If we had first taken the mean of the expert response first, and then transformed that estimate to obtain our model coefficient, we would no longer be able to generate a variance, as we would have only one estimate.

For each covariate of interest, we used $\left({\hat{\bar{\beta }}}_{jk},SE\left({\hat{\bar{\beta }}}_{jk}\right)\right)$ to define a normal prior distribution in our prediction models. Each prior was based on a maximum of 20 responses, but item-level nonresponse reduced the number of responses to varying degrees (see Table A2 for individual response counts). Due to the small sample sizes, we ignored the potential covariance between the coefficients, resulting in a variance-covariance matrix that is only non-zero on the diagonal. This is different from the methods evaluated in West et al. (2019) that utilize historical data to generate priors. For those methods, including the historical method replicated in our results, estimated covariances were generated from the existing historical data.

Table A2 in the online appendix provides the prior information, $\left({\hat{\bar{\beta }}}_{jk},SE\left({\hat{\bar{\beta }}}_{jk}\right)\right)$, for each covariate included in the propensity models, provided that there were at least three contributing respondents. Further, an Excel spreadsheet available in the online supplementary material provides a template for estimating these priors for the survey items in the propensity model. For demonstration purposes, simulated data are included in the table, including missing cells, which would occur should an expert not respond to a particular question.

### Methods for Predicting and Evaluating Response Propensities

Each of the five NSFG quarters of interest (Quarters 16 through 20, representing June 2015 - September 2016) were analyzed independently to introduce replication in our analysis. First, we used the expert opinions to generate the prior distributions for the response propensity model coefficients as described above. These priors were used for all five quarters.

We generated our “target” prediction at the case level for each of the five evaluation quarters by fitting a discrete time-to-event logistic regression model using the predictors identified in the backward selection model discussed in Section 3.2 to all contact attempt records from that quarter. This allowed us to estimate a “final” probability of responding to the screener interview at the last contact attempt for each case. Because this model uses all available information for a given quarter, we consider this the benchmark against which the prediction methods under evaluation will be compared. Table 1 below shows the ROC-AUC values when all contact attempt records were used to predict final response.

These model fit statistics reflect the in-sample performance of the models and demonstrate that the variable selection procedure from West et al. (2019), where these statistics are extracted from, yielded a reasonable list of predictors for our target response propensity. From that point, we are concerned with the case-level differences from the target propensity that the different methods produce.

Then, we generated daily predictions of response propensity based on contact history data accumulated prior to each day. Our baseline predictions came from the model using only accumulating current round paradata. Our proposed predictions came from the model that also incorporated prior information from expert opinion. Additionally, we included predictions that incorporate prior information from historical data, as presented in West et al. (2019). In that paper, the authors found that the historical data method performed the best in their application. We include the historical data method here so we can understand how well the expert elicitation method performs when compared to both the “current data only” method and one of the historical data methods evaluated in West et al. (2019).

Prediction of daily response propensity for each of these three methods is carried out just as it would have been if the approach were to be employed during data collection. For each of the five quarters of interest, we use the accumulated contact attempt record information (with a screener response indicator for each record) up to day *d* to estimate the coefficients for the discrete time logistic regression model for that data collection period. Then we use those coefficients to predict the response propensity at the next contact attempt for all cases who were nonrespondents on day *d*. We repeat this for each day of data collection from Day 7 to Day 84.

Using only the current quarter of paradata, the response propensity, ${\hat{p}}_{id}$, was modeled as follows:

(4) $${\hat{p}}_{id}=\hat{p}\left(y_{id}=1|X_{id}\right)=\frac{\exp\left({\text{Σ}}_{v=0}^{V}{\hat{\beta }}_{v}X_{idv}\right)}{1+\exp\left({\text{Σ}}_{v=0}^{V}{\hat{\beta }}_{v}X_{idv}\right)}$$

where *y*_*id*_ is the response status for the *i*^*th*^ case after a contact attempt on the *d* ^*th*^ day, and *X*_*id*_ is the set of predictors *v* for the *i*^*th*^ case after the *d*^*th*^ day. These predictors may be fixed (e.g., geographic predictors) or time-varying (e.g., prior contact status). The ${\hat{\beta }}_{v}$ are estimated coefficients for the *X*_*idv*_ predictors. They are estimated from the likelihood in equation (5) based on the contact attempt records that have been accumulated through day *d*.

(5) $$L\left({\hat{\beta }}_{0},\ldots ,{\hat{\beta }}_{v}\right)=\prod_{i=1}^{n}\prod_{j=1}^{d}{\left(\frac{\exp\left(\sum_{v=0}^{V}{\hat{\beta }}_{v}X_{idv}\right)}{1+\exp\left(\sum_{v=0}^{V}{\hat{\beta }}_{v}X_{idv}\right)}\right)}^{y_{id}}{\left(1-\left(\frac{\exp\left(\sum_{v=0}^{V}{\hat{\beta }}_{v}X_{idv}\right)}{1+\exp\left(\sum_{v=0}^{V}{\hat{\beta }}_{v}X_{idv}\right)}\right)\right)}^{\left(1-y_{id}\right)}$$

The only difference between the target prediction and the baseline, current-data only method is the time at which the prediction is made. For the target predictions, all contact attempt records from a given quarter are used (*d* is after the last contact attempt is made in a given quarter); for the baseline method, only data accumulated through day *d* are used.

In a Bayesian setting (Gelman et al. 2013), the likelihood matches the frequentist formulation. The only estimated parameters in this expression are the ${\hat{\beta }}_{v}$, and so these are the parameters for which priors are defined. As described in Section 3.4, we assumed a normal distribution, $\beta_{v}∼N\left(\mu_{v},\sigma_{v}^{2}\right)$, for our priors with the mean and variance based on our expert elicitation procedure. The posterior multiplies the prior over the parameters in the likelihood to combine the information, as shown in equation (6):

(6) $$pos\left({\hat{\beta }}_{0},\ldots ,{\hat{\beta }}_{v}\right)=\prod_{i=1}^{n}\prod_{j=1}^{d}\left[{\left(\frac{\exp\left(\sum_{v=0}^{V}{\hat{\beta }}_{v}X_{idv}\right)}{1+\exp\left(\sum_{v=0}^{V}{\hat{\beta }}_{v}X_{idv}\right)}\right)}^{y_{id}}{\left(1-\left(\frac{\exp\left(\sum_{v=0}^{V}{\hat{\beta }}_{v}X_{idv}\right)}{1+\exp\left(\sum_{v=0}^{V}{\hat{\beta }}_{v}X_{idv}\right)}\right)\right)}^{\left(1-y_{id}\right)}\right]\;\times \prod_{v=0}^{v}\frac{1}{\sqrt{2\pi \sigma_{v}^{2}}}exp\left(-\frac{1}{2}{\left(\frac{\beta_{v}-\mu_{v}}{\sigma_{v}}\right)}^{2}\right)$$

In the Bayesian version of the prediction, it is clear that the priors add additional information to the prediction. This can be beneficial when the likelihood is based on very sparse data, or partial data that are not representative of the full data collection process, both of which occur earlier in the data collection process. Code in the SAS 9.4 programming language that can be used to carry out these predictions is available in the online supplementary materials.

For each method, we will compare predictions for each contact attempt on each day of the data collection quarter to the “target” predictions (based on all cumulative data) in order to generate daily estimates of the bias and root mean squared error (RMSE) for the predictions. The mean daily bias for the *m*^*th*^ method is defined as:

(7) $$B^{m}=\frac{1}{n}\sum_{i=1}^{n}\left({\hat{\rho }}_{i}^{m}-\rho_{i}\right)$$

and the daily RMSE for the *m*^*th*^ method is defined as:

(8) $$RMSE^{m}=\sqrt{\frac{1}{n}\sum_{i=1}^{n}{\left({\hat{\rho }}_{i}^{m}-\rho_{i}\right)}^{2}}$$

We then summarized those estimates using boxplots for three different parts of data collection: early (day 7 – 30), middle (day 31 – 60), and late (day 61 – 84).

The end-of-data-collection response propensity is not the only possible target, but this choice does allow us to evaluate whether the use of Bayesian approaches with informative priors can reduce error in the predictions of response propensity at a given contact attempt versus using only current round paradata. Additionally, we will be able to evaluate whether the use of expert opinion (in the absence of historical data) can perform similarly to the historical data, were it available.

## Results

### Descriptive Statistics for Selected Priors

We first wanted to understand if ISR experts have different expectations than Census experts, potentially due to the varying familiarity with NSFG or simply being a part of a different survey organization. We also collected information about the experts’ length of experience with survey data collection, thinking opinion may vary with length of experience and more experienced managers may provide more useful information. We then examined distributions of the individual experts’ betas, generated using Equations (1) and (2) above, by organization and experience level. Here we provide examples of these distributions to illustrate similarities and differences in the provided opinions. Due to the small sample sizes, we do not provide tests of significance with respect to these differences. Instead, we are interested in the means and general trends of the expert opinion by category in order to understand, at a high level, if different types of experts provide different information.

**Figure 1:**
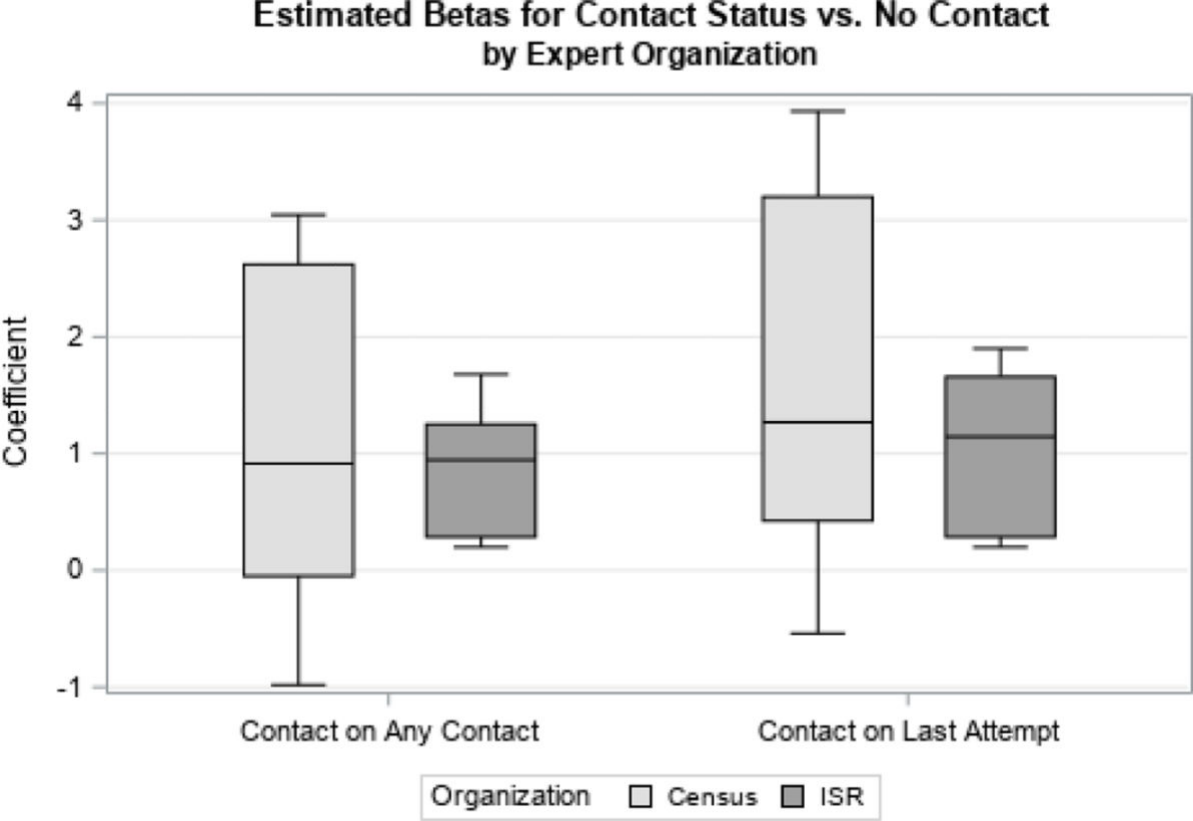
Coefficients for Contact Status by Organization

**Figure 2:**
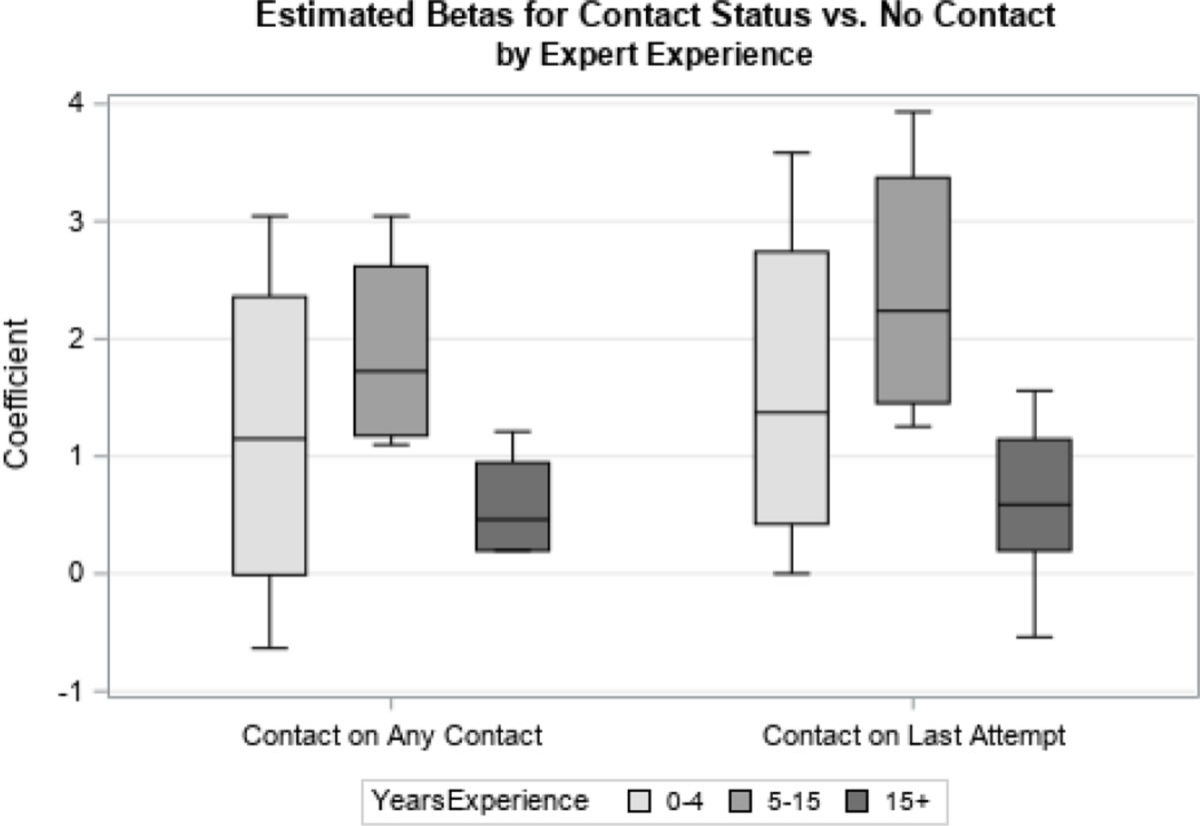
Coefficients for Contact Status by Experience

**Figure 3:**
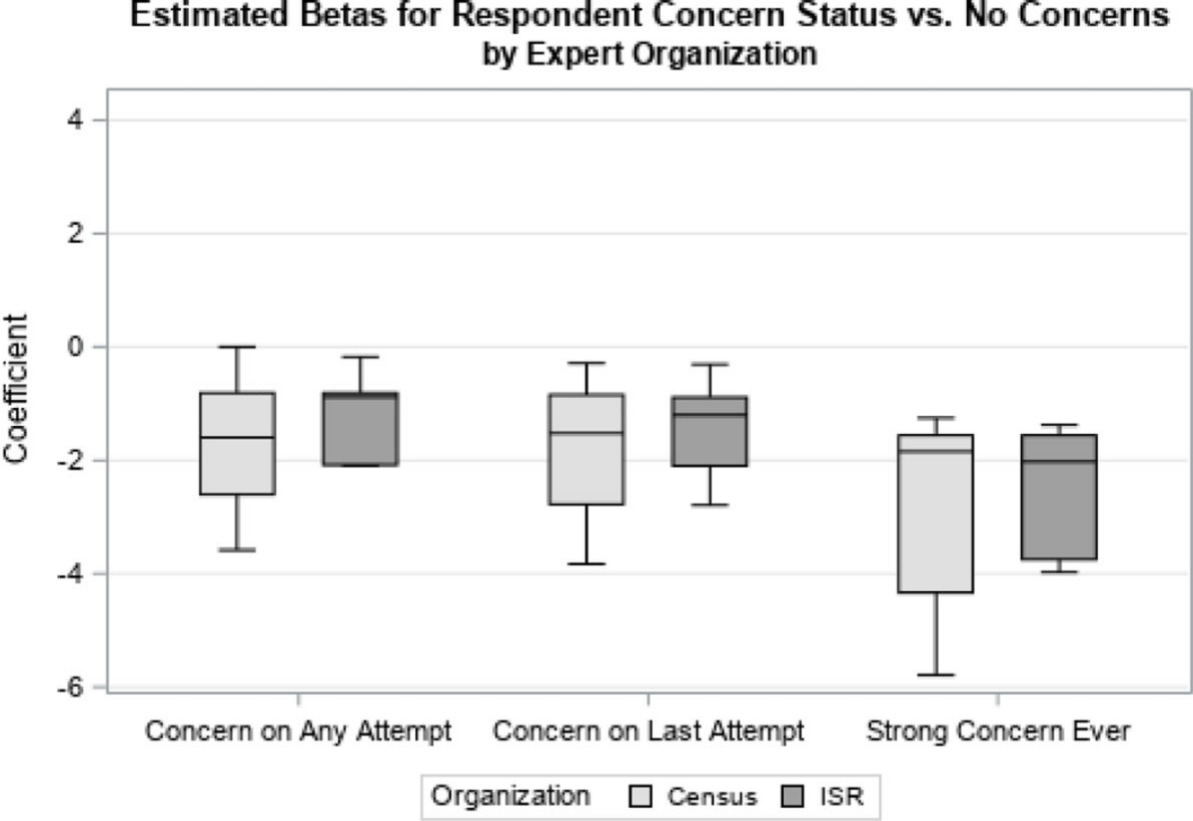
Coefficients for Expressed Concerns by Organization

**Figure 4:**
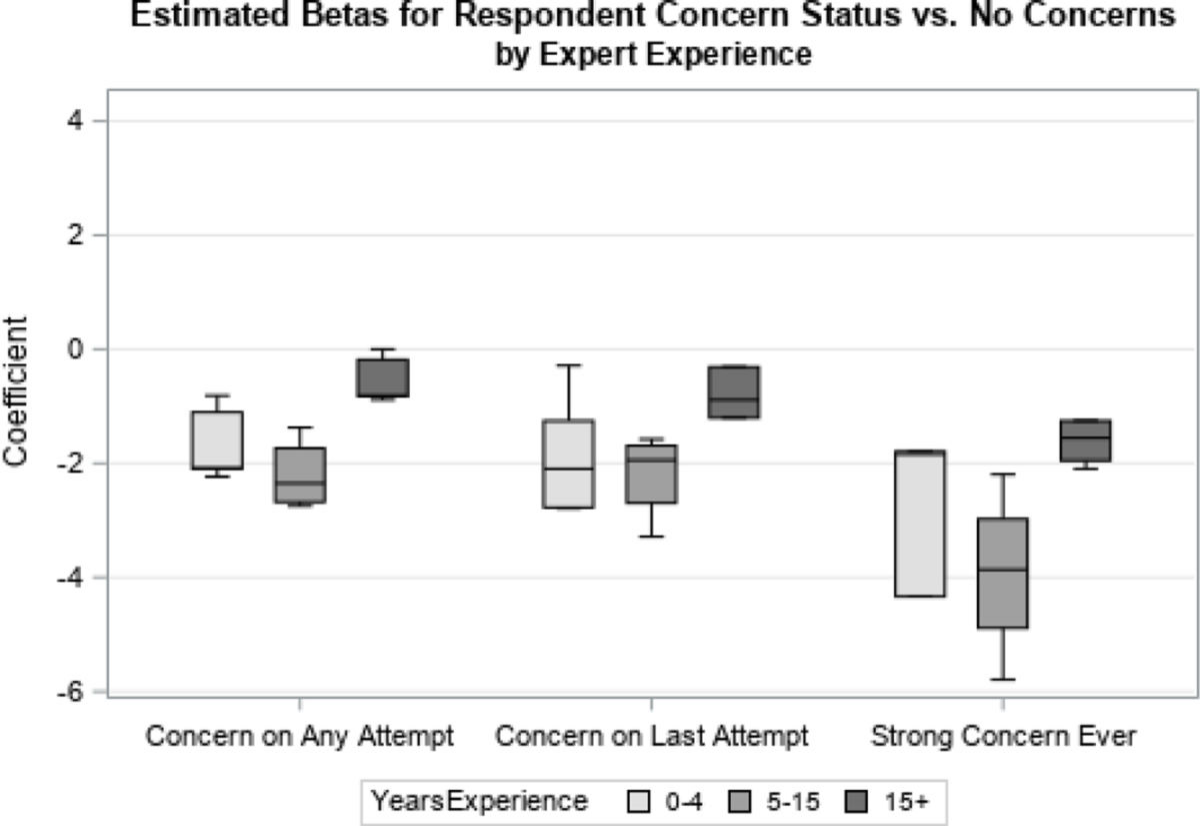
Coefficients for Expressed Concerns by Experience

We first examined distributions of coefficients related to two time-varying covariates, Contact Status and Concerns Status. Contact Status had three possible response categories: if there was ever contact with the sample member, contact on the previous attempt, or if there had never been contact with the respondent, which was used as the reference category. Concerns Status had four possible response categories: if concerns were ever expressed by the sample member, if concerns were expressed on the previous visit, if strong concerns were ever expressed, or if no concerns were ever expressed (the reference category). We looked at how responses differed by organization (Figures 1 and 3) and level of experience (Figures 2 and 4).

For both variables, we found largely the same results. There were no large differences found in the point estimate for the priors by survey organization, shown in Figures 1 and 3.

When examining the priors by level of experience (Figures 2 and 4), interviewers with 0–4 or 5–10 years of experience generated similar point estimates for the betas, while experts with fifteen or more years of experience showed differences with respect to the point estimates. Specifically, experts with 15 or more years of experience appear to perceive, on average, that any one covariate has less of an impact on response propensity than do experts with less experience.

**Figure 5:**
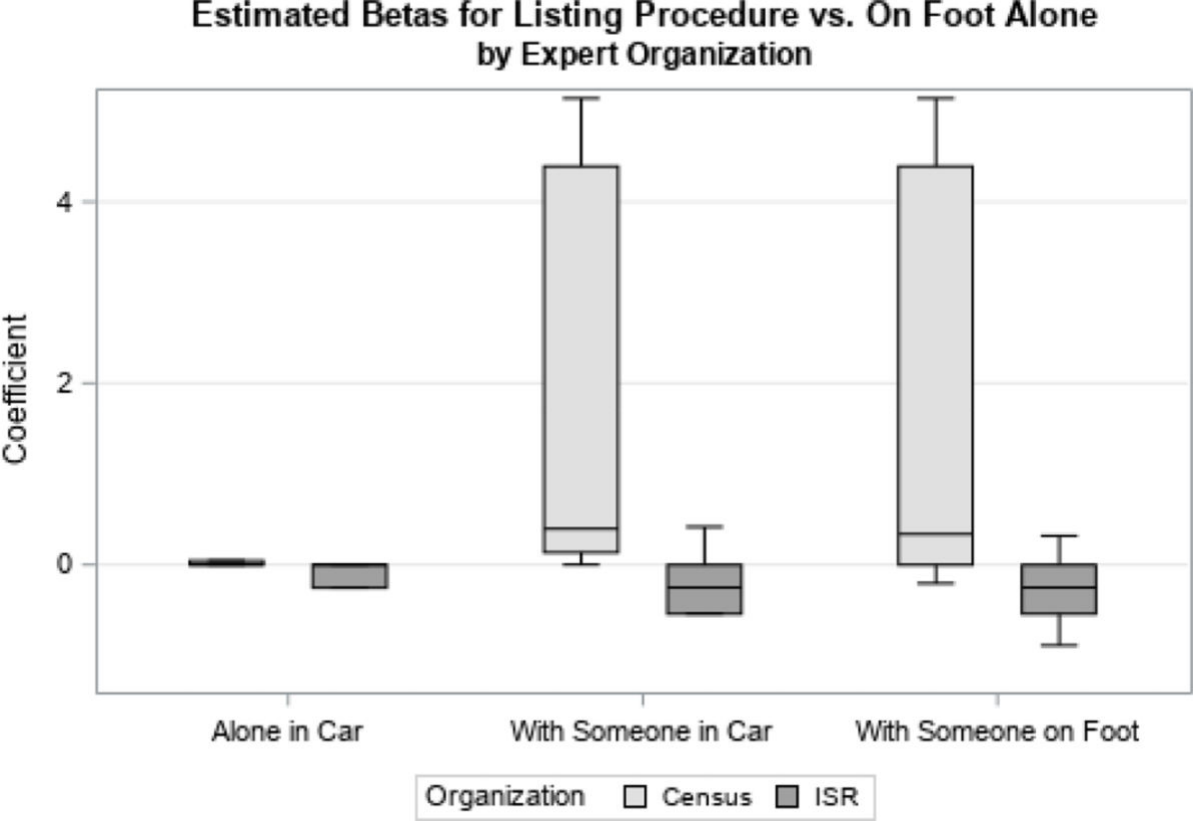
Estimated Betas for Listing Procedure by Organization

Other questionnaire items showed more clear differences between the survey organizations. Figure 5 shows the effect of various types of listing procedures on response propensity, versus listing alone on foot. Here, there are not only differences in the means by survey organization, particularly for listing in a car with another person and on foot with another person, but the means are in the opposite directions from the reference category, and the Census Bureau estimates are highly variable compared to estimates from ISR. In this particular case, feedback showed that Census Bureau experts did not see a link between listing method and response propensity, resulting in highly variable responses. We discuss the additional expert feedback that we received on the survey more in Section 5.

**Figure 6:**
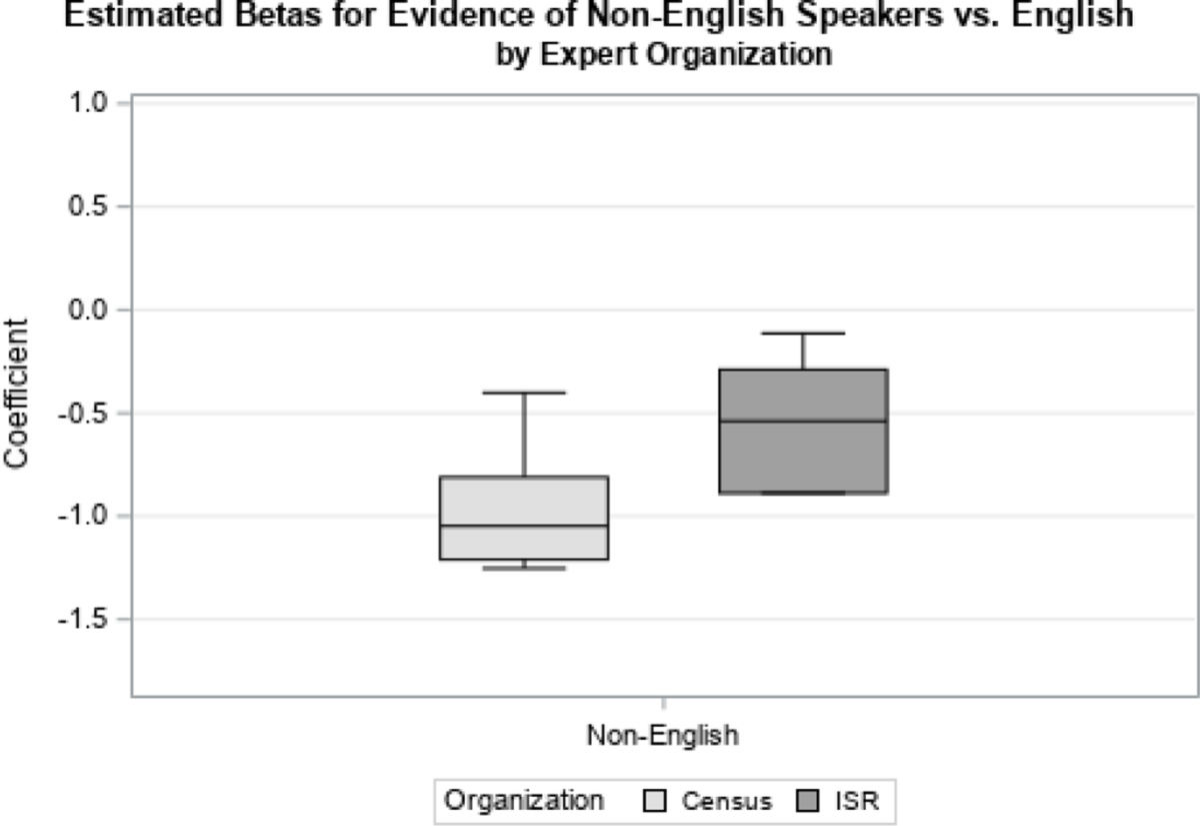
Estimated Betas for Likely Non-English Speaker by Organization

Figure 6 displays the distributions of the betas by survey organization for the effect of evidence of a language other than English being spoken at home. Here, Census Bureau experts feel that evidence has a more negative effect on response propensity than ISR experts do. This may have to do with differences in the availability of bilingual interviewers or language specialists.

Understanding these similarities and differences is important for selecting the most appropriate experts to interview. Depending on the survey of interest, it might be more important to select interviewers with specific skill sets, such as language specialties. It may also affect which questions are included on the questionnaire, or which priors are actually used in the prediction model. In the case of listing procedure, the feedback obtained might suggest ignoring the prior information for some or all of the experts, and either using an uninformative prior or dropping the variable from the model.

### Comparison of Methods

**Figure 7:**
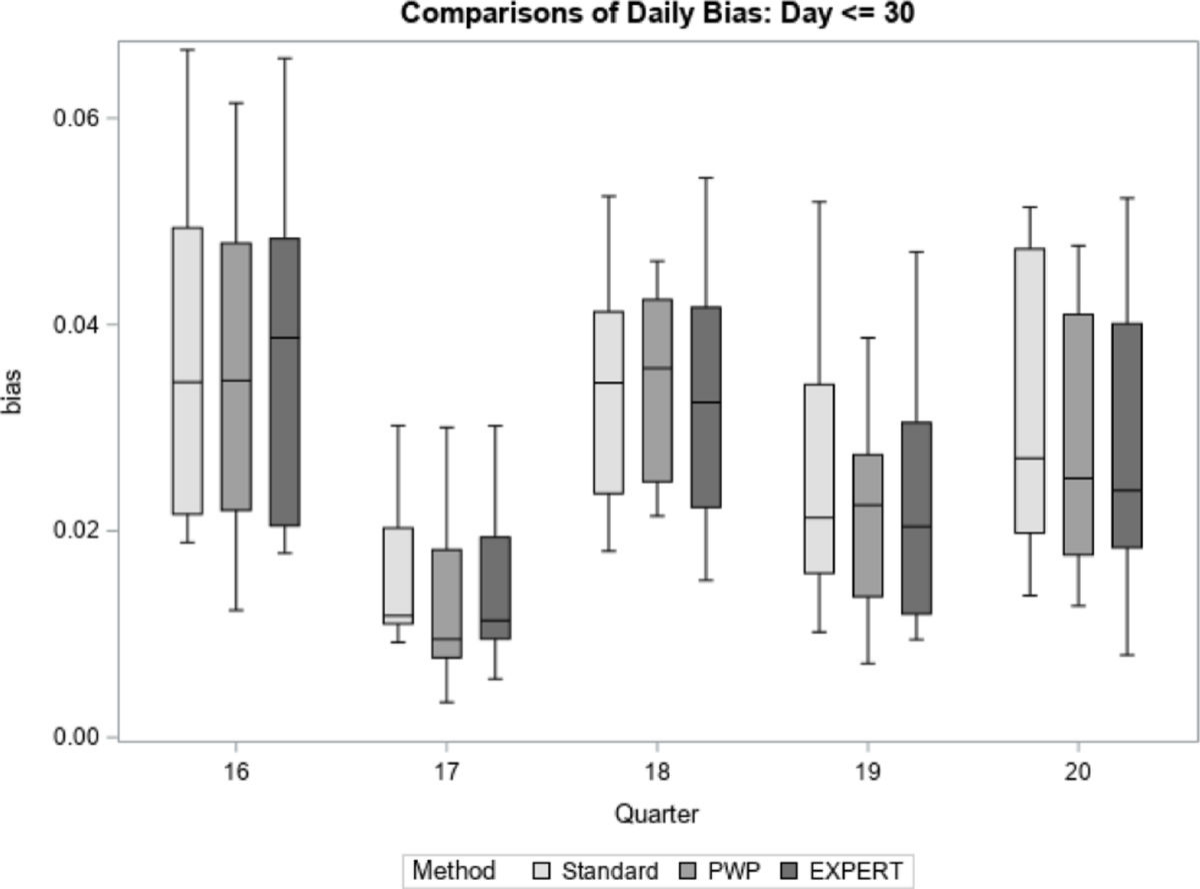
Bias in Response Propensities by Quarter (Early)

**Figure 8:**
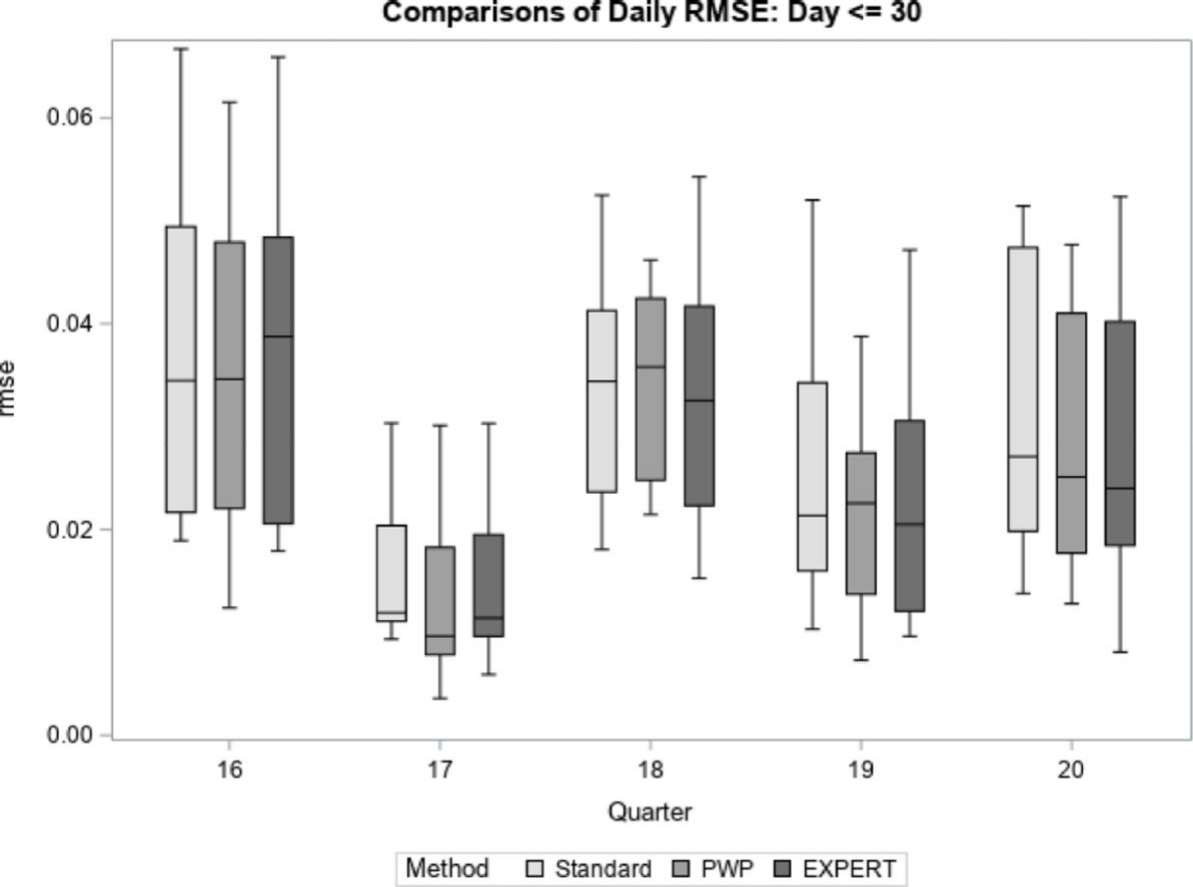
RMSE of Response Propensities by Quarter (Early)

**Figure 9:**
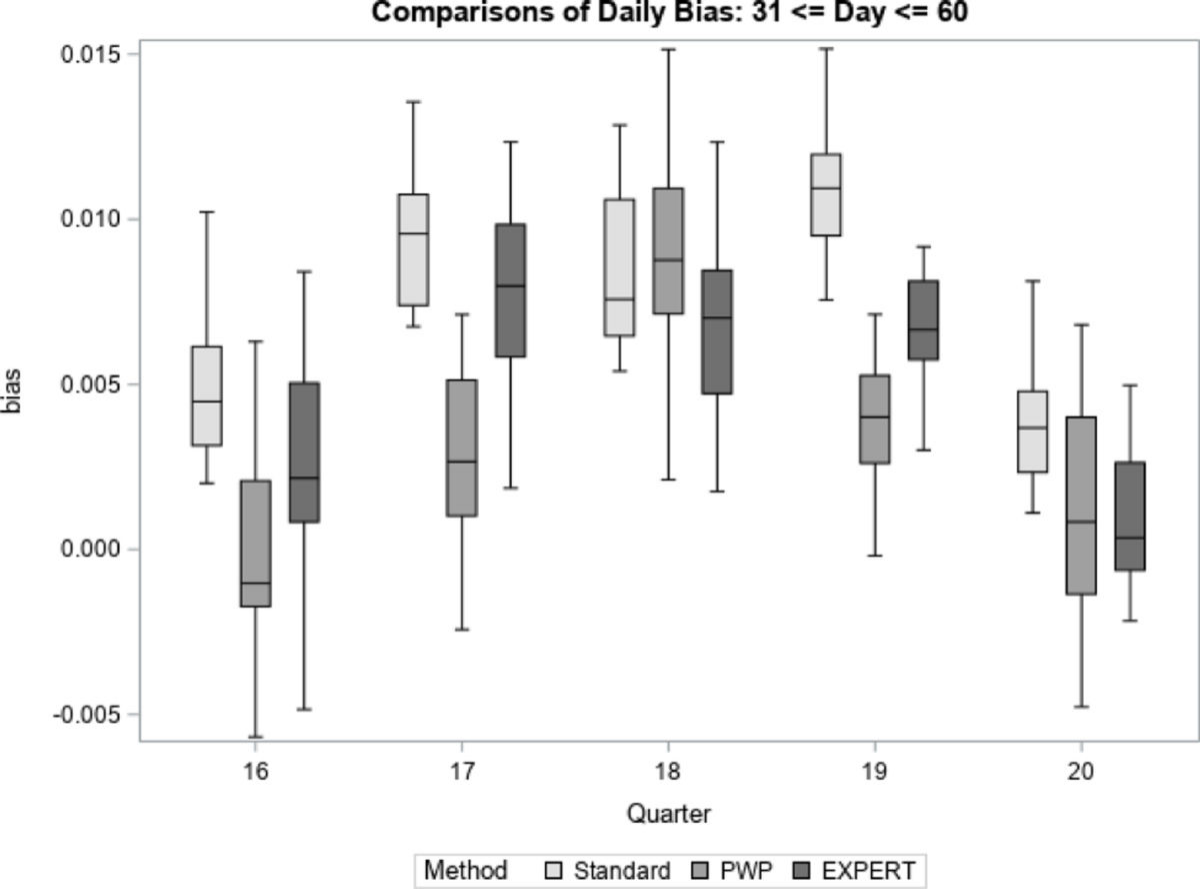
Bias in Response Propensities by Quarter (Mid)

**Figure 10:**
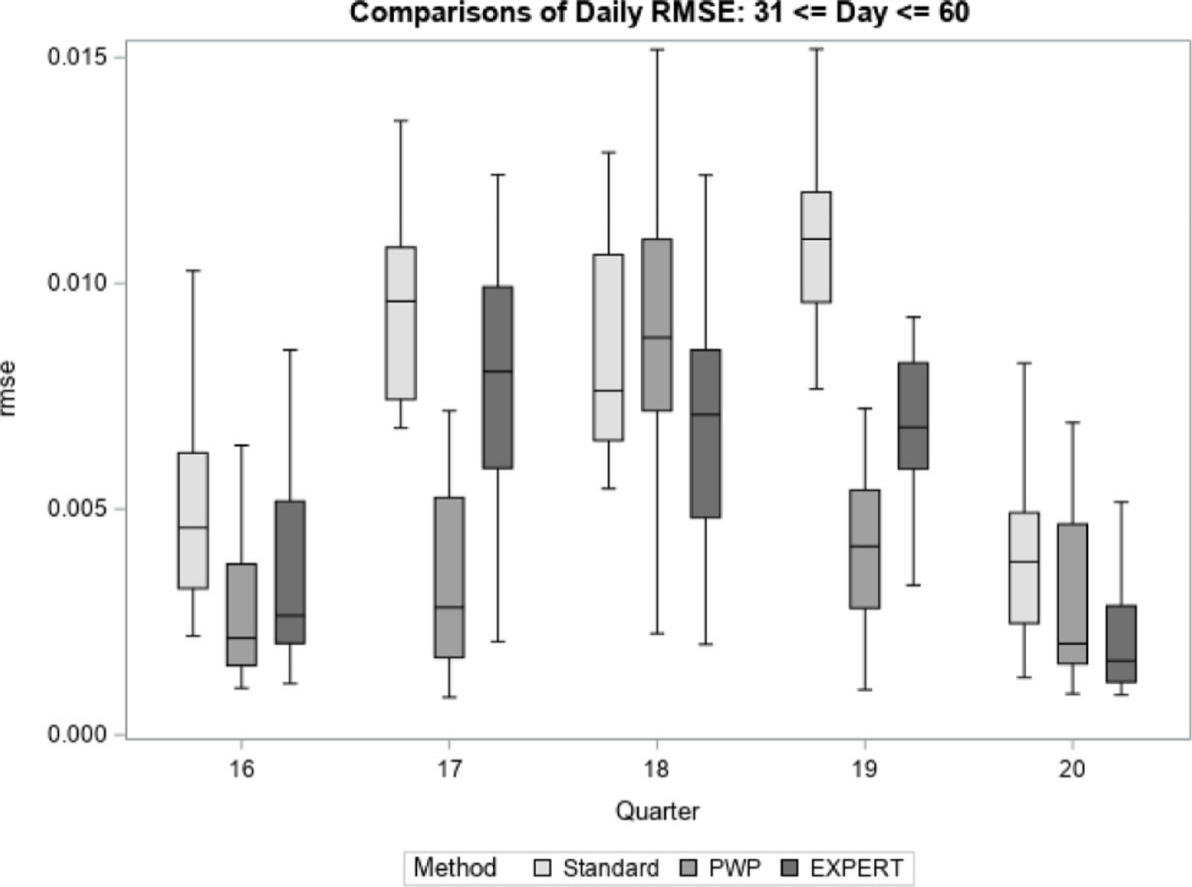
RMSE of Response Propensities by Quarter (Mid)

**Figure 11:**
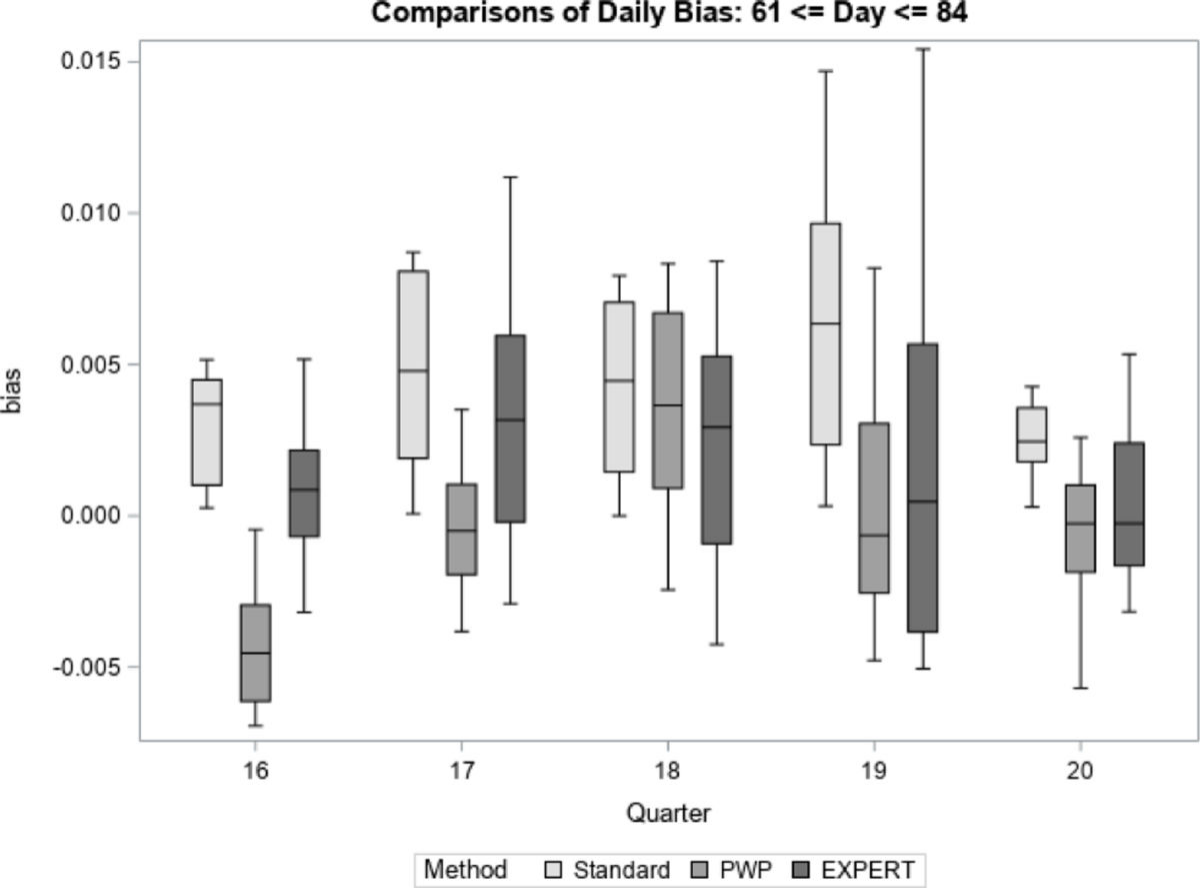
Bias in Response Propensities by Quarter (Late)

**Figure 12:**
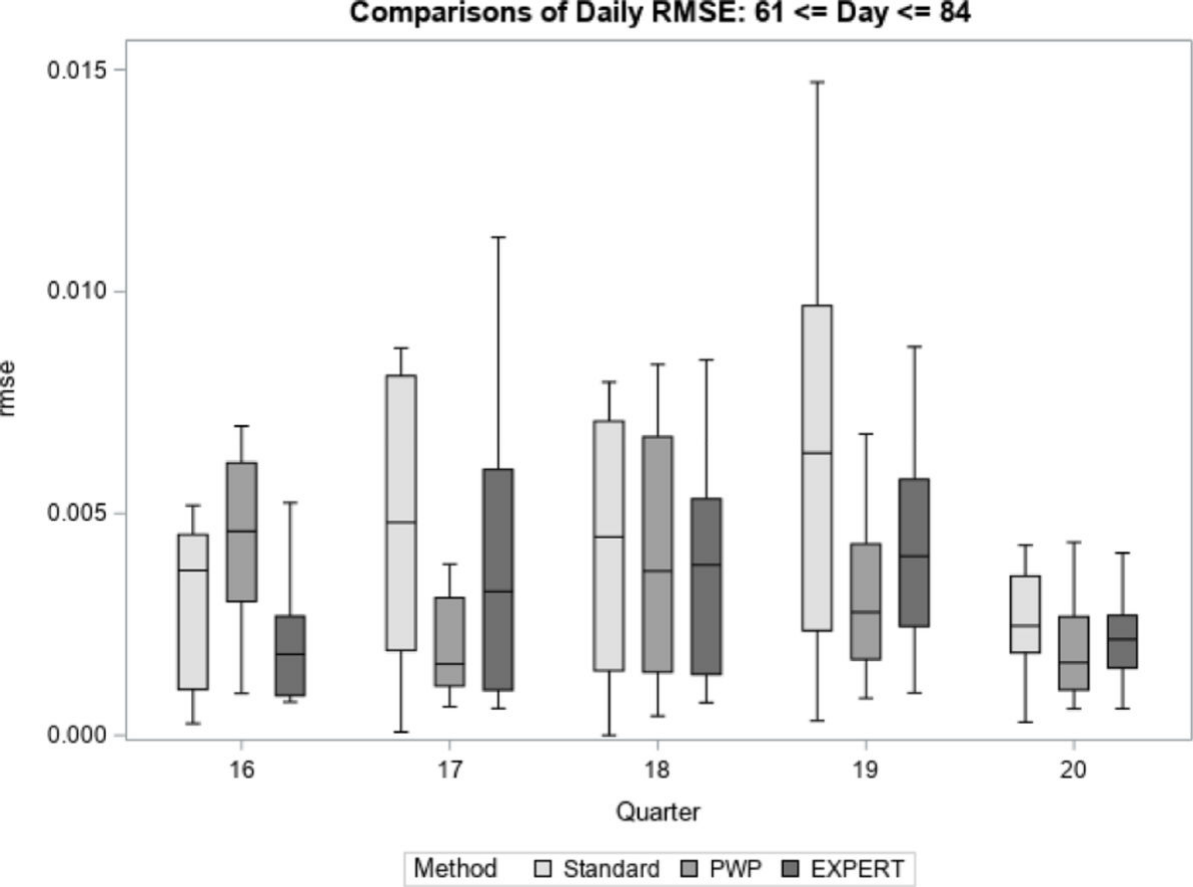
RMSE of Response Propensities by Quarter (Late)

For each quarter, we treated the final prediction of response propensity, based on all accumulated contact data for the quarter, as the unbiased “target” prediction of response propensity. For each method, we then generate daily estimates of bias and RMSE with respect to the target prediction. Figures 7 to 12 display the performance of the Bayesian method using expert elicitation (EXPERT) to the current data-only method (Standard) and the precision-weighted prior Bayesian method (PWP) from West et al. (2019) that incorporates historical data. Our primary interest was to evaluate whether predictions generated using priors derived from expert opinion would be of higher quality than those generated using current data only, assuming historical data were not available for use. However, we were also interested in how the priors from expert opinion perform versus priors from historical data, which were evaluated in West et al. (2019). Because this was a retrospective analysis, we were able to examine both of these questions. Figures 7, 9 and 11 present the summarized distributions of estimated bias, while Figures 8, 10, and 12 present the summarized distributions of estimated RMSE.

Figures 7 and 8 focus on the early portion of data collection, from day 7 through day 30 (24 days). For each quarter, the 24 daily estimates of bias (Figure 7) or RMSE (Figure 8) were summarized using box plots. Early in data collection, the expert elicitation (EXPERT) method has a small but inconsistent effect on the bias and RMSE versus the standard method. For example, in quarters 19 and 20, the EXPERT method results in mean, median, and intraquartile ranges of both the bias and RMSE of the predictions that are slightly closer to zero than the Standard method, signifying an improvement. However, in quarter 16, the EXPERT method performs worse than the Standard method with respect to the mean and median values of bias and RMSE, and delivers no improvement in quarter 17. Overall, however, neither the PWP nor the EXPERT method offer consistent improvement over the Standard method early in data collection.

Figures 9 and 10 below represent the middle portion of data collection from day 31 to day 60. Beginning on day 31, there are noticeable reductions in the bias and RMSE of predictions for the EXPERT method. In all five quarters, the central tendencies of both the bias and the RMSE, as well as the intraquartile range, are shifted towards zero versus the Standard method. Further, in quarter 19, neither of the metrics have interquartile ranges that overlap between the Standard and EXPERT methods. For the most part, the PWP method continues to perform at least as well as the EXPERT method on measures of bias and RMSE, though the EXPERT method is certainly competitive, particularly in quarters 18 and 20. Here, unlike in the early portion of data collection, there is a clear benefit to using priors from expert elicitation if historical data are not available.

During the final third of data collection, shown below in Figures 11 and 12, we continue to see that the EXPERT method leads to reduced measures of bias and RMSE versus the Standard method. These improvements are generally smaller than those found in Figures 9 and 10. Over the course of data collection, as more data are accumulated, it is likely that the Standard method improves in its ability to predict response, leading to smaller differences between the Bayesian methods and the Standard method. Additionally, it is more mixed as to whether the historical method or the expert opinion method is superior.

These results show that for this application, the PWP method results in the most consistent improvements in bias and RMSE of predictions of response propensity. However, the results also show that, in the absence of historical information, predictions that incorporate expert opinion still generally outperform the standard method, and can be a useful way to improve predictions of response propensity during data collection for the purposes of an RSD.

### Feedback from Survey Experts on Prior Questionnaire Development

Within two weeks of receiving questionnaire responses, we elicited feedback from experts in order to uncover issues with the questionnaire and identify potential areas for improvement. The experts had feedback in three main areas: the concepts identified in the questionnaire, how those concepts were translated into variables and categorical subgroups, and the lack of anchor points throughout the questionnaire.

The design of the questionnaire was driven by the variables available from the frame or from paradata. However, the concepts measured in the questionnaire did not always match concepts considered by the recruited experts. In our questionnaire, the experts provided two examples of this issue. In one instance, the predictive covariates from existing data sources were not meaningful concepts for survey managers. Mail Delivery Point Type is a categorical variable providing information on how mail is delivered to an address. This variable comes from the commercially available data and has several different categories that were significant in the variable selection model discussed in Section 3.2. However, when we included this variable (and all significant categories) on the expert questionnaire, only three out of 20 survey managers responded for any of the categories. During debriefing, survey managers explained that they did not have any experiential evidence that there was a relationship between response propensity and mail delivery. As a result, the survey managers generally declined to provide information for this concept.

On the other hand, survey managers explained that they do make use of concepts that were not included on the questionnaire. When providing feedback, one survey manager from the Census Bureau mentioned “perceived safety in a neighborhood” as a predictor of response propensity. In this case, this category was not included on the questionnaire because it was not a significant predictor in the response propensity model described in Section 3.2. It may be worthwhile to elicit information about predictors suggested by field experts, in order to capture information about predictors the experts find informative or predictive. This would allow confirmation that those particular items do not offer more explanatory power than the items retained from the propensity model.

In addition to defining meaningful concepts, it was also important to translate each concept into a variable that generated informative predictions, to the extent possible. This included determining whether a variable should be categorical or continuous, and, if categorical, how to define subgroups. Again, we found two clear examples of this issue. First, there were some instances where the categories that we provided in the expert questionnaire were not the same as those in the baseline model. As an example, age of householder, sourced from the sampling frame, was defined in the current model as having four categories: 18 – 44; 45 – 59; 60+; and Missing. In the questionnaire, we only included three categories to simplify the response options: Under 50; 50+; and Missing. Age of the householder is provided on the sampling frame as a continuous variable, so in this instance, the different classifications posed no issues for generating predictions of response propensity. However, if the questionnaire included categories that were not able to be derived from the existing frame or paradata, the priors derived from expert information would not easily translate to covariates in the existing data.

The survey experts also suggested that the functional form of some of our variables was not ideal. For example, on the questionnaire, we asked the experts to predict the change in attempt-level response rates for every $10,000 increase in household income over the median. At least one expert suggested that the relationship was likely not linear, and a better way to elicit opinion might be categorical, such as using quartiles of household income. This would better represent what the experts suggested, which was that the top and bottom quartiles of household income would have a lower attempt-level response rate than those in the middle two quartiles.

The experts also provided feedback regarding anchor points. In designing the questionnaire, we made a conscious decision to only include the overall attempt-level response rate, 24%, in the introduction, leaving it up to respondents to generate all subgroup level response rates. This was primarily to avoid generating anchoring bias among the survey expert responses. However, while survey managers were comfortable ordering different subgroups of a variable, from highest to lowest predicted response rates, and even defining relative differences, they were less comfortable defining an initial response rate for one category, in order to then provide response rates that reflected the subgroup ordering and relative differences. We found evidence of this in the response data itself. Survey managers provided responses for nearly all questions, but on occasion, the predicted response rate ranges varied significantly (e.g., one manager might have all subgroup response rates in a range of 20% to 40%, while another would provide responses in a range of 60% or 80%). One survey manager suggested providing an anchor point for one subgroup in the categorical variable, from which they could then provide the relative differences for the remainder of the subgroups. We provided an overall anchoring point in order to facilitate estimates of effect levels. The 24% value acts as an “intercept” attempt-level response rate, from which specific categories of the questionnaire deviate. However, we did not provide any category-level anchor points in an effort to avoid anchoring bias. There was a concern that if we provided the overall attempt level response rate (24%) in addition to an anchor point for one of the categories, the experts would focus on the relationships between categorical response rates and the overall response rates. For example, had we provided the 24% overall attempt-level response rate, and a response rate of 35% for female respondents, the expert may ignore their own expertise to provide a response rate around 13% in order to have the categorical response rates roughly match the overall attempt-level response rate. Our goal was to provide the minimum necessary amount of background information to allow the experts to use their own judgement to the fullest extent possible.

## Discussion

We hypothesized that in the absence of historical survey data, survey researchers would be able to generate priors from the experiences of survey managers that lead to improved predictions of response propensity over those made from just the data available for the current round of data collection. The results of this study demonstrate that eliciting expert opinion is a useful way to generate priors and improve prediction of response propensities. Particularly after the first month of the NSFG data collection process, priors generated from expert opinion resulted in predictions of next-contact response propensity with both lower bias and RMSE than predictions based on only current round data. One potential explanation for why the Bayesian methods did not improve the predictions in the first month of data collection is that the early experience in any quarter is highly variable. That is, in Bayesian terms, the likelihood varies from quarter to quarter in the first few weeks. The observed data are somewhat more stable after 30 days, but do not normally align with the final model until near 60 days into the quarter. Hence, it is during that interval – i.e. after the first 30 days but before the 60th day of the quarter – that the prior information is most useful.

This prior elicitation process is significantly more involved than building models from existing historical data. Developing a questionnaire, conducting data collection with survey experts, aggregating and organizing the response data, and generating priors may be time consuming, particularly as the number of covariates increases. As a result, eliciting expert opinion for generating priors may not always be the ideal solution. In our experience, the large majority of the time and effort was spent on the initial development of the questionnaire. We would expect changes, adaptations, and future implementations to require much less effort. Experts themselves spent, on average, less than an hour on the actual survey. Assuming a pay rate of $50 per hour, the actual elicitation portion of the survey would cost roughly $1,000. We can imagine numerous applications where this type of expenditure would be worth this cost, as in the case where a new survey has a specific target population that may not have coefficients well-estimated by the published literature. Further, this method may be useful for mathematically incorporating expert opinion into predictions of response rates for budgetary purposes, sample sizes, and power calculations. Given the high costs of face-to-face data collection, improved response propensity predictions may help data collection managers make better decisions in an adaptive or responsive design framework. Evaluating of the ability of predictions based on such an approach to improve data collection outcomes is an interesting direction for future research. We are currently pursuing experimental work in this area.

Through the process of designing and implementing the questionnaire, debriefing the survey managers, and analyzing the collected data, we identified four areas survey researchers should consider when developing and implementing expert elicitation surveys. These areas include the selection of concepts for inclusion into the survey; the translation of those concepts into covariates and/or categories; the potential need for anchor points for categorical covariates; and lastly, the selection of experts for the survey. Attention to these areas will lead to information from experts that is more helpful for generating priors, which are ultimately combined with current data to generate posterior predictions of response propensity.

For this particular questionnaire, through debriefings and response analysis, we observed several opportunities for improvement in the design process for expert surveys. Mindful selection of concepts and the subsequent translation of categorical variables will help experts provide more informative prior expectations. By working with experts to determine which data fields on the frame and in the paradata effectively translate to concepts used by survey managers, the value of the elicited information may increase. Additionally, it may uncover concepts used by survey managers when developing ad hoc expectations for response propensities that are not currently provided by data systems. There may be an opportunity then for expert opinion to motivate a modification of existing systems, either by appending an additional piece of information from the survey frame (if available), or capturing this concept in paradata, potentially through interviewer observations.

In order for experts to provide opinions on attempt level response rates for a survey, particularly when they are unfamiliar with the exact topic questionnaire, it may be helpful to provide context to the survey managers about general attempt-level response rates, or even provide an anchor point for one category of a variable. Providing an anchor point for a particular subgroup may be a reasonable solution to this issue, but it may increase anchoring bias in the remainder of the experts’ responses. Additionally, in the case of categorical covariates in a logistic regression, it may not be absolutely critical. Generating priors requires constructing odds ratios, using one subgroup as a reference category. Because of this, odds ratios focus on the relative difference between a category of interest and a baseline category more than point estimates of response propensities provided by the survey managers. As a result, if the ordering and relative differences are accurate, that may be sufficient for generating relatively useful priors.

Associated with this is the fact that continuous variables were queried about on a linear scale, while the logistic regression modeling assumes a log-odds scale. For categorical variables this transformation is straightforward, since there is only a fixed set of options for the categorical variable to take; for continuous covariates, however, extrapolations outside of the specific values considered lead to different predictions. Thus, if an expert suggests that an additional contact attempt increasing the probability of a successful contact from 5% from a 24% baseline, this yields a beta parameter of 0.26; thus five contact attempts increase the odds of contact to 54%, instead of the 49% on the linear scale, and to 81% after transformation from the log-odds scale for 10 contact attempts, vs. 74% on the original linear scale. Hossack, Hayes and Barry (2017) have proposed eliciting priors at a series of quantiles of the continuous predictor values in order to better approximate the log-odds transformation; we leave this as a future extension.

An iterative process to address these issues is difficult to carry out without collaboration with the targeted experts and may not be possible in all situations. However, if it is possible to first validate a questionnaire with some experts, keeping in mind the potential biases like overconfidence and anchoring biases, the resulting questionnaire may have more predictive power. Similarly, the SHELF method, proposed by O’Hagan (2019) relies on a significant amount of interaction with the experts throughout the elicitation process in order to elicit a probability distribution form each expert. While this method can be highly informative, providing both a point estimate and a measure of uncertainty for each expert’s opinion, the number of items in our questionnaire would not have allowed for this level of individual interaction.

We also used the variability in the point estimates across our sample of experts to determine the variability in the prior distribution. This simplified the task of constructing the prior, since the experts were required only to supply point estimates, not estimates of uncertainty. This required a relatively large sample size of experts compared to many such elicitation studies. It also allowed us to take advantage of the Central Limit Theorem to utilize a normally-distributed prior, which in turn allowed more direct comparisons with West et al. (2019); alternatively, more heavy-tailed priors (e.g., t-distributions with small degrees of freedom) could be used. We did not rescale the prior to account for this sample size; one could construct a prior based on a “pseudo-sample size” of *m* by multiplying $SE\left({\hat{\bar{\beta }}}_{jk}\right)$ in (4) by $\sqrt{n/m}$ (that is, standard deviation of the arithmetic mean by the square root of *m* rather than the square root of the actual number of respondents). Alternatively, one could elicit estimates of uncertainty as well as point estimates from the expert sample, and use information for both the direct elicitation and the sampling variability to construct the variance of the prior; we leave this to future research.

A limitation of our approach is that we used historical data to determine the key covariates to include in our survey of experts. We did this in order to make a fair comparison with historical data in our analysis, but in practice one might at best have data available from other studies with greater or lesser degrees of similarity. Indeed, one might have no historical data whatsoever from which to build a propensity model, in which case one would have to rely on experts’ opinion about potentially predictive items to develop an effective model for response propensity. As noted in Section 5, querying experts for the key covariates may have advantages over model selection, even if historical data is available from similar studies.

Finally, it is important to elicit expert opinion from appropriate individuals, based on the survey characteristics. Experts at ISR were identified through discussions with survey managers to identify appropriate individuals. At the Census Bureau, we worked with senior leadership in the Field Directorate to identify the two “most knowledgeable” survey managers in each of the six regional offices. This provided geographic coverage over the entire country and, we hoped, significant experience in demographic surveys that could be translated into priors for response propensity prediction. We did not include any other requirements in our identification of survey managers for interview. After collecting responses, we found that survey experience ranged anywhere from ‘0–4 years’ to ‘15 or more years’, and we found potential correlations between experience and predictions of attempt-level response rates predictions for some covariates. Due to the small sample size, we cannot conclude that these correlations are meaningful. However, it is useful to consider whether additional requirements would be useful when identifying experts. Relevant experience, either with respect to survey topic (e.g., health, education, etc.), operations (e.g., multimode vs. in-person interviewer-administered), or other characteristics, may lead to more informative expert opinion for incorporating into priors.

## Supplementary Material

Supplemental Excel spreadsheet

Supplemental material, questionnaire

Supplemental material, code

## Figures and Tables

**Table 1:** Model Fit Statistics for In-Sample Predictions of Response, 5 Evaluation Quarters

	Q16	Q17	Q18	Q19	Q20
ROC-AUC	0.711	0.682	0.661	0.690	0.654
Nagelkerke-Pseudo R^2^	0.143	0.115	0.089	0.130	0.086

## Appendix

**Table A1:** Significant predictors of screener response propensity in the final discrete time logit model for call-level data from the eight most recent quarters, after applying backward selection (n = 119,981 calls; Nagelkerke pseudo R-squared = 0.09; AUC = 0.66).

Predictor	Coefficient	Standard Error
Intercept	−2.56	0.32
Mail Delivery Point Type: Missing	0.08	0.03
Mail Delivery Point Type: A	0.03	0.02
Mail Delivery Point Type: B	−0.04	0.03
Mail Delivery Point Type: C	−0.09	0.03
Interviewer-Judged Eligibility: Missing	2.46	0.10
Interviewer-Judged Eligibility: No	0.63	0.07
Segment Listed: Car Alone	0.03	0.02
PSU Type: Non Self-Representing	0.06	0.03
PSU Type: Self-Representing (Not Largest 3 MSAs)	0.03	0.03
Previous Call: Contact	3.97	0.28
Previous Call: Different Window	−0.12	0.02
Previous Call: Building Ever Locked	0.32	0.05
Previous Call: Building Locked	2.16	0.14
Previous Call: Strong Concerns Expressed	0.26	0.04
Previous Call: No Contact	2.26	0.13
Previous Call: Other Contact, No Concerns Expressed	−1.35	0.25
Previous Call: Concerns Expressed	−1.58	0.26
Previous Call: Soft Appointment	−1.03	0.30
Previous Call: Call Window Sun.-Thurs. 6pm-10pm	0.07	0.03
Previous Call: Call Window Fri.-Sat. 6pm-10pm	0.08	0.02
No Access Problems in Segment	−0.05	0.02
Evidence of Other Languages (not Spanish)	−0.09	0.03
Census Division: G	−0.14	0.03
Census Division: B	−0.32	0.03
Census Division: D	−0.22	0.03
Census Division: H	−0.24	0.03
Census Division: C	−0.20	0.03
Census Division: F	−0.27	0.04
Census Division: E	−0.20	0.03
Census Division: A	−0.19	0.04
Contacts: None	−0.68	0.24
Contacts: 1	−0.54	0.22
Contacts: 2 to 4	−0.42	0.19
Segment Domain: <10% Black, <10% Hispanic	−0.04	0.02
Segment Domain: >10% Black, <10% Hispanic	−0.04	0.02
Segment Domain: <10% Black, >10% Hispanic	0.01	0.03
Percentage of Segment Non-Eligible (Census Data)	−0.01	<0.01
Interviewer-Estimated Segment Eligibility Rate	−0.55	0.12
Interviewer-Estimated Household Eligible	−0.09	0.02
Segment Type: All Residential	0.04	0.02
Log(Number of Calls Made)	−0.60	0.03
Log(Number of Calls Made) x No. Prev. Contacts	−0.04	0.01
CML* HoH Age: 35–64	−0.12	0.02
CML Adult Count: Missing	−0.13	0.04
CML Adult Count: 1	−0.09	0.03
CML Adult Count: 2	0.01	0.03
CML Asian in HH: Missing	0.21	0.04
CML Asian in HH: No	0.20	0.05
CML HoH Gender: Missing	−0.03	0.02
CML HoH Gender: Female	−0.01	0.02
CML HoH Income: $35k-$70k	0.12	0.02
CML HoH Income: less than $35k	0.14	0.02
CML HH Own/Rent: Missing	−0.06	0.03
CML HH Own/Rent: Owned	−0.02	0.02
CML Age of 2^nd^ Person: Missing	−0.13	0.03
CML Age of 2^nd^ Person: 18–44	−0.15	0.03
No Respondent Comments	0.08	0.04
Non-Contacts: None	−0.51	0.08
Non-Contacts: 1	−0.25	0.05
Non-Contacts: 2–4	−0.03	0.03
Occupancy Rate of PSU	−0.26	0.10
Respondent Other Concerns	0.18	0.06
Physical Impediment to Housing Unit: Locked	−0.35	0.03
Day of Quarter	0.01	<0.01
Respondent Concerns Expressed: None	−1.25	0.15
Respondent Concerns Expressed: Once	0.15	0.09
Single Family Home / Townhome	−0.22	0.03
Structure with 2–9 Units	−0.29	0.04
Structure with 10+ Units	−0.21	0.04
Respondent Concern: Survey Voluntary?	−0.46	0.15
Respondent Concern: Too Old	0.60	0.15

* CML denotes that the variable came from a commercial data source.

**Table A2:** Normal Prior Definitions, $\left({\hat{\bar{\beta }}}_{jk},SE\left({\hat{\bar{\beta }}}_{jk}\right)\right)$, for all predictors included in the NSFG response propensity model described in Section 3.2. The table notes which categories served as reference categories in the prior generation process, and also notes how many responses (out of a maximum of 20) that we received for each category.

	All Respondents (max n = 20)		
	
Questions and Categories	Count of Responses	Mean Beta	StdErr Beta
*Gender of Primary Householder (vs. Male)*			
Female	20	0.336	0.063
Missing	14	−0.465	0.257
*Age of Primary Householder (vs. 50 or Over)*			
< 50	20	−0.370	0.108
Missing	15	−0.831	0.293
*Number of Adults in HH (vs. 2 or More)*			
1	20	0.066	0.198
Missing	12	−0.732	0.219
*Race/Ethnicity of Primary Householder (vs. Asian)*			
White	18	0.532	0.121
Black	18	−0.031	0.173
Hispanic	18	−0.118	0.112
Other	13	−0.348	0.233
Missing	12	−0.326	0.292
Household Income Effect			
+$10,000	17	0.466	0.235
*Masked Census Division (vs. Region I)*			
G	14	0.020	0.129
B	14	−0.205	0.138
D	14	0.041	0.141
H	14	0.060	0.161
C	14	0.133	0.170
F	15	0.294	0.150
E	15	0.057	0.145
A	16	−0.050	0.192
*Race/Ethnicity Sampling Domain (vs. > 10% Black, > 10% Hispanic)*			
< 10% Black, < 10% Hispanic	16	0.696	0.202
> 10% Black, < 10% Hispanic	16	0.535	0.132
< 10% Black, > 10% Hispanic	16	0.364	0.143
*Access Problems (vs. Other)*			
Locked Buildings/Gated Communities	19	−0.687	0.190
Seasonal Hazardous Conditions	18	−0.418	0.153
Unimproved Roads	17	0.267	0.164
None	10	1.091	0.189
*Evidence of Non-English Languages (vs. No)*			
Yes	15	−0.725	0.163
*Neighborhood Age Effect*			
10 years older than national average	17	0.520	0.099
*Occupancy Rate Effect*			
10% increase in occupancy rates	16	0.187	0.170
*PSU Type (vs. Major Metropolitan Area)*			
Minor Metropolitan Area	18	0.155	0.155
Not Metropolitan	17	0.398	0.158
*Listing Procedure (vs. On Foot Alone)*			
On Foot With Someone	11	0.787	0.607
In a Car Alone	11	−0.066	0.135
In a Car With Someone	11	0.795	0.614
*Structure Type (vs. Other)*			
Single Family Home	5	1.172	0.567
Structure with 2–9 Units	5	0.788	0.602
Structure with 10+ Units	5	0.600	0.617
Mobile Home	5	0.728	0.462
*Delivery Type (vs. Other)*			
Curbline	3	0.917	0.590
Neighborhood Delivery Collection Box	3	0.199	0.289
Central	3	0.069	0.384
Missing	3	0.000	0.000
*Physical Impediments (vs. Other)*			
Locked Entrance	19	−0.096	0.206
Doorperson or Gatekeeper	19	−0.627	0.117
Access controlled via Intercom	19	−0.371	0.106
None	14	1.076	0.155
*Attempt-Level Concerns Expressed (vs. No Concerns)*			
Concerns Expressed on Previous Attempt	17	−1.347	0.434
Concerns Expressed Not on Previous but Prior Attempt	17	−1.451	0.244
Strong Concerns Ever Expressed	15	−2.228	0.593
*Attempt-Level Contact (vs. Never Contacted)*			
Contacted at Previous Attempt	15	1.367	0.329
Not Previous but Prior Contact	15	1.009	0.298
*Contact Observations (vs. Other)*			
Ever Said „Too Old“	14	−0.532	0.336
Comment re: Voluntary Nature of Survey	17	0.335	0.489
Any Other Comments	14	0.118	0.182
Never Made Comment	13	0.325	0.205
*Day of Field Period Effect*			
Change in RR for Each Day of Field Period	12	0.213	0.078
*Call Window (vs. Weekday Day)*			
Weekday Evening	19	1.203	0.193
Weekend Day	19	1.052	0.166
Weekend Evening	19	0.426	0.220
*Ever Requested Call-Back/Soft Appointment (vs. No)*			
Yes	18	0.564	0.339
*Concatct Attempt Effect*			
Change in RR for Each Additional Contact	17	−0.058	0.109
*Contact*Contact Interaction Effect*			
Change in RR for Each Add’l Call*Contact	13	0.177	0.228
