# Supplementary material for: What Do You Think? Using Expert Opinion to Improve Predictions of Response Propensity Under a Bayesian Framework
Source: Methoden Daten Anal. Author manuscript; Available in PMC 2021 Jun 3. (PMC8174793; doi:10.12758/mda.2020.05)
Supplement: Supplemental material, questionnaire [file NIHMS1694538-supplement-Supplemental_material__questionnaire.pdf]

The following questions include several **socio-demographic factors** obtained for a large proportion (but not all) of the sample from a commercial source. The commercial data provide household characteristics (e.g. number of adults in household) and person-level characteristics (e.g. gender) for multiple persons. Here, we ask about subgroups defined by the first person (i.e. “Person 1”) listed in the commercial data. For each factor, a separate estimate of the **call-level response rate** for cases that did not have that variable available on the commercial data (i.e. “Missing Data”) is needed. For each subgroup defined by these factors, please indicate the expected **call-level response rate**. If you do not have any sense of response rates for a particular group, please write “NE” (i.e. “no estimate”) in the box. Do not worry about making sure that the overall call-level response rate would be 24% from your subgroup estimates.

Factor	Subgroup of Selected Respondent	Expected Call-Level Response Rate
Gender of Person 1	Male	
	Female	
	Missing Data	
Age of Person 1	Under 50	
	50+	
	Missing Data	
Number of Adults in Household	1	
	2+	
	Missing Data	
Race/Ethnicity of Person 1	White	
	Black	
	Hispanic	
	Asian	
	Other	
	Missing Data	

Factor		Expected Change in Call-Level Response Rate (Use Negative Sign for Expected Decreases)
Estimated Household Income	How much does a $10,000 increase in median income raise or lower response rates?	
	Missing Data	

The next section asks about expected response rates for different **types of neighborhoods**. These data are available from the sampling frame (Census-based Area Characteristics) or from interviewer observations.

Please enter a percentage as a number between 0 and 100 without the percent sign. For example, 25% would be entered as “25”.

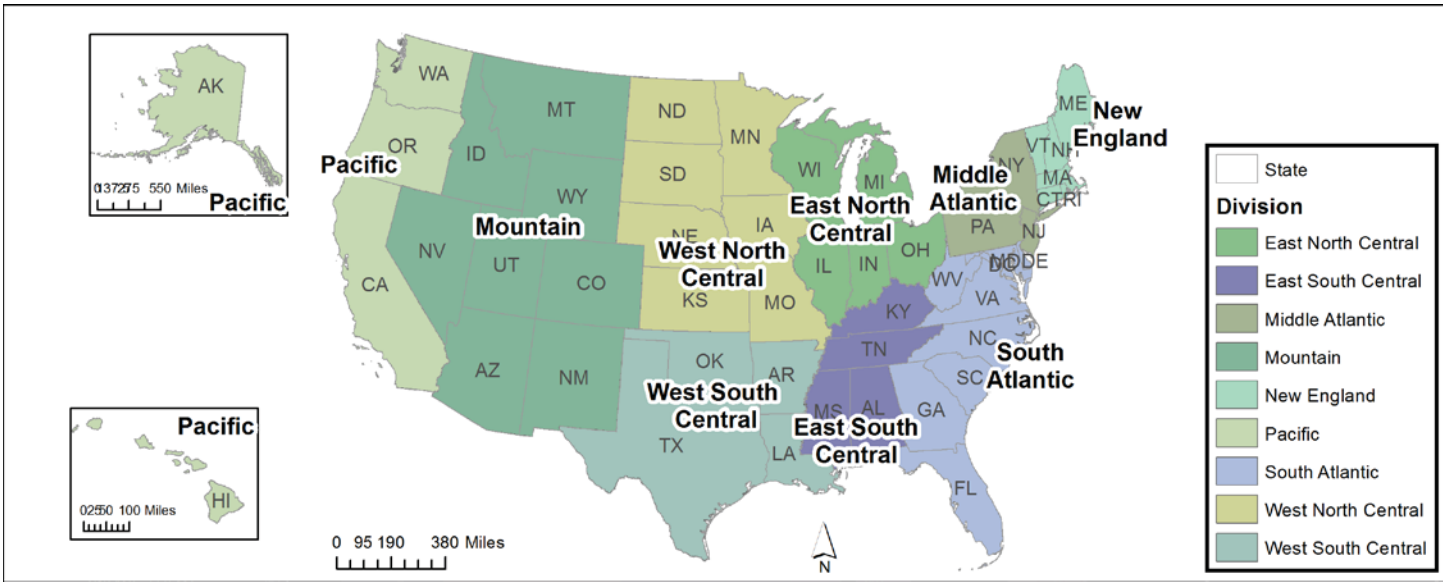

**Factor**	**Neighborhood Characteristic**	**Expected Call-Level Response Rate**
**Census Division** (Census Data)	New England	
	Middle Atlantic	
	South Atlantic	
	East North Central	
	East South Central	
	West North Central	
	West South Central	
	Mountain	
	Pacific	
**Race/Ethnicity Sampling Domains** (Census Data)	<10% Black, <10% Hispanic	
	>10% Black, <10% Hispanic	
	<10% Black, >10% Hispanic	
	>10% Black, >10% Hispanic	

Factor	Neighborhood Characteristic	Expected Call-Level Response Rate
**Access Problems** (Interviewer Observations)	Locked Buildings/Gated Communities	
	Seasonal Hazardous Conditions	
	Unimproved Roads	
	None	
**Evidence of non-English Languages** (Interviewer Observations)	Yes	
	No	

Factor	Neighborhood Characteristic	Expected Change in Call-Level Response Rate (Use Negative Sign for Expected Decreases)
**Neighborhood Age Distribution** (Census Data)	How much does response rate change if average age is 10 years older than national average?	
**Occupancy Rate** (Census Data)	How much does a 10 percentage point increase in the occupancy rate increase response rates?	

Factor	Neighborhood Characteristic	Expected Call-Level Response Rate
**PSU Type** (Census Data)	Major Metropolitan Area	
	Minor Metropolitan Area	
	Not Metropolitan	
**Listing Procedure** (Interviewer Obs)	On foot alone	
	On foot with someone	
	In a car alone	
	In a car with someone	

The next section is about specific **features of housing units**. These are based upon interviewer observations.

Please enter a percentage as a number between 0 and 100 without the percent sign. For example, 25% would be entered as “25”.

Factor	Housing Unit Type	Expected Call-Level Response Rate
**Structure Type**	Single Family Home	
	Structure with 2 to 9 units	
	Structure with 10+ units	
	Mobile Home	
	Other	
**Delivery Type**	Curbline	
	Neighborhood Delivery Collection Box Unit (NDCBU)	
	Central	
	Other	
	Missing	
**Physical Impediments**	Locked Entrance	
	Doorperson or other gatekeeper	
	Access controlled via intercom	
	None	

This final section looks at the impact of different paradata on response rates. Many of these are specific features of call attempts. For example, “Resistance on Prior Attempt” is a paradata element indicating that the prior attempt experienced resistance. We are asking for your estimate of how this each feature will impact the response rate of the next attempt.

Please enter a percentage as a number between 0 and 100 without the percent sign. For example, 25% would be entered as “25”.

Factor	Paradata-Defined Group	Expected Call-Level Response Rate
**Attempt-Level Resistance**	Resistance on previous attempt	
	No resistance on previous attempt, but resistance on prior attempts	
	Ever maximum resistance or “Hard Refusal”	
	Never Resistant	
**Attempt-Level Contact**	Contacted at previous attempt	
	No contact at previous attempt, but contact at prior attempt	
	Never contacted	
**Contact Observations**	Ever said “too old”	
	Comment related to voluntary nature of survey	
	Made other comment	
	Never made a comment	

Factor		Expected Change in Call-Level Response Rate (Use Negative Sign for Expected Decreases)
**Day of Field Period**	How much does response rate increase or decrease for each day of the field period?	

Factor		Expected Call-Level Response Rate
Call Window	Weekday Day	
	Weekday Evening	
	Weekend Day	
	Weekend Evening	
Ever Requested Soft Appointment or General Callback Time?	Yes	
	No	

Factor		Expected Change in Call-Level Response Rate (Use Negative Sign for Expected Decreases)
Attempt Characteristic	Each Call Attempt (i.e. how much does each call attempt lower the response rate)?	
	Number of calls interacted with number of previous contacts	

Finally, we have some simple background questions for you.

How many years of experience do you have working on surveys?

☐ 0 to 4 years ☐ 5 to 9 years ☐ 10 to 15 years ☐ 15 or more years

How old are you?

☐ 18-29 ☐ 30-39 ☐ 40-49 ☐ 50-59 ☐ 60+

What is your gender?

☐ Female ☐ Male

Thank you for your help!
