# Supplementary material for: What Do You Think? Using Expert Opinion to Improve Predictions of Response Propensity Under a Bayesian Framework
Source: Methoden Daten Anal. Author manuscript; Available in PMC 2021 Jun 3. (PMC8174793; doi:10.12758/mda.2020.05)
Supplement: Supplemental material, code [file NIHMS1694538-supplement-Supplemental_material__code.pdf]

```
/*****************************************************************************/
/* Coffey, S., West, B.T., Wagner, J., Elliott, M.R. (2019).                 */
/* Code adapted from Brady West                                              */
/*                                                                           */
/* Sample Code to Estimate Daily Response Propensity Two Ways for Evalution  */
/*    Method 1: Using Current Accumulating Data Only                         */
/*    Method 2: Setting Priors and Obtaining Predictions from a Bayesian Posterior    */
/*                                                                           */
/* This code assumes an attempt level file (multiple records per case) where each     */
/* each attempt is dated. This allows a response propensity model to be estimated     */
/* daily, based on the most recent contact attempt information. Each model covariate  */
/* should be a column in the dataset, and the covariates can be fixed or time-varying.*/
/*                                                                           */
/* In our paper, we include many covariates & generate predictions for five quarters. */
/* For this sample code, we include 5 predictors, and use just one quarter.  */
/*****************************************************************************/

libname rp_est "\your\data\location";

/**********************************************************************************************
/* Set priors for use in Bayesian prediction. Input priors as macro vars for easy management.
/* Use one set of macro statements to assign the point estimate of the priors, and another for varianc
/**********************************************************************************************
data _null_;
set est;   /*input data here actually irrelevant as it is only to assign macro vars*/
    /* estimates */
    call symput('b0mean',0);
    call symput('b1mean',0.136);
    call symput('b2mean',-0.324);
    call symput('b3mean',-1.25);
    call symput('b4mean',0.760);
    call symput('b5mean',-0.02);
    /* variances */
    call symput('b0var',10);
    call symput('b1var',0.026);
    call symput('b2var',0.015);
    call symput('b3var',0.124);
    call symput('b4var',0.325);
    call symput('b5var',0.0001);
run;

/**********************************************************************************************
/* Estimate daily predictions of response propensity using accumulating paradata - no priors
/* Retain last prediction of response propensity for each case after all attempts are recorded.
/* Generate final "target" response propensity using all accumulated paradata for the current quarter
/**********************************************************************************************

/* run the final model first, with no priors (generates final parameter estimates and predicted respon
    proc logistic data= rp_est.input;
        class var1(ref="1") var2(ref="3") var3(ref="2") / param = ref;
        model comp(event="1") = var1 var2 var3
                                var4 var1*var4 / rsq lackfit;
        output out=finalout p=final_phat stdxbeta=stdxbeta xbeta=xbeta;
        ods output ParameterEstimates = final_rp_est;
    run;
/* extract final resposne propensities for each case */
```

```
        proc sort data=finalout; by caseid descending callday; run;
        proc sort data=finalout nodupkey (keep= caseid callday final_phat); by caseid; run;

/* macro for daily estimates using current data only or Bayesian method */
%macro byday(s, f);
    %do i = &s. to &f.;
        data daily;
        set rp_est.input;
            if callday gt &i. then delete;
            runday = &i.;
            method = "current";
        run;
        proc logistic data= daily;
            class var1(ref="1") var2(ref="3") var3(ref="2") / param = ref;
            model comp(event="1") = var1 var2 var3
                                    var4 var1*var4 / rsq lackfit;
            output out=dailyout p=daily_phat stdxbeta=stdxbeta xbeta=xbeta;
            ods output ParameterEstimates = daily_rp_est;
        run;
        /* extract final response propensities for each case on each day*/
        proc sort data=dailyout; by caseid descending callday; run;
        proc sort data=dailyout nodupkey out= daily_d&i. (keep= caseid method callday runday daily_rp_
        /* append the most up-to-date response propensity for each case on each day */
        %if &i. = &s. %then %do;
            data current_phat;
            set daily_d&i.
            run;
        %end;
        %if &i. ne &s. %then %do;
            data current_phat;
            set current_phat daily_d&i.;
            run;
        %end;
    %end;
    /* order daily predictions */
    proc sort data=current_phat; by caseid runday; run;

    /* append final reponse propensity to daily estimate for each case */
    proc sql;
    create table rp_est.current_phats as
    select a.*, b.final_phat
    from current_phat as a
    left join final_phat as b
    on a.caseid = b.caseid
    order by a.caseid, a.callday
    ;quit;

    /* Bayesian modeling on each day */
    %macro byday(s, f);
       %do i=&s. %to &f.; /*days of interest in data collection period*/

            data pred_day&i.;
            set rp_est.input;
                if callday gt &i. then delete;
                runday = &i.;
            run;
```

```
/*Bayesian approach to fitting model to data from up to that day*/
proc mcmc data=pred_day&i. ntu=100 nmc=5000 nthin=5 propcov=quanew plots=none
            outpost=outtest;
    parms ( b0 b1 b2 b3 b4 b5 ) 0;

    prior b0 ~ normal (&b0mean,var=&b0var);
    prior b1 ~ normal (&b1mean,var=&b1var);
    prior b2 ~ normal (&b2mean,var=&b2var);
    prior b3 ~ normal (&b3mean,var=&b3var);
    prior b4 ~ normal (&b4mean,var=&b4var);
    prior b5 ~ normal (&b5mean,var=&b5var);

    p=logistic(b0 + b1*var1 + b2*var2 + b3*var3 + b4*var4 + b5*(var1_var4) ;
    model iwcompflag ~ bern(p);

    ods output postsumint=post&i;

run;

/*extract vectors of draws of 5 coefficients, transpose for matrix multiplication*/
data outtest2 (keep = b0--b5);
set outtest;
run;

proc transpose data = outtest2 out = betas;
run;

data betas (drop = _NAME_);
set betas;
run;

/* obtain microdata from today's subset, in specific order of coefficients */
data subsub (keep = caseid callday one var1 var2 var3 var4 interact );
set outtest_day&i.;
    one = 1;
    interact = var1*var4;
run;

/* sort by ID and contact number, to maintain correct order */
proc sort data = subsub;
    by caseid callday;
run;

/* make sure microdata are in correct order, and only keep relevant variables */
data subsub2 (keep = one var1 var2 var3 var4 interact );
retain one var1 var2 var3 var4 interact ;
set subsub;
run;

/* compute logits based on each draw using IML */
proc iml;
    dsNames = {subsub2 betas};
    MatNames = dsNames;
    do i = 1 to ncol(dsNames);
        use (dsNames[i]);
```

```
            read all var _NUM_ into X;
            call valset(MatNames[i], X);
            close (dsNames[i]);
        end;
        show names;
        scores = subsub2 * betas;
        create out2 from scores;
        append from scores;
        close out2;
    quit;

    /* compute 1000 draws of p-hat for each contact attempt on each case on this day, and then
    data out3 (keep = daily_rp_est);
    set out2;
        array p[1000];
        array l[1000] col1--col1000;
        do i = 1 to 1000;
            p[i] = exp(l[i]) / (1+exp(l[i]));
        end;

        daily_rp_est = mean(of p1--p1000);
    run;

    /* simple merge back into microdata possible due to explicit sorting of cases by ID and co
    data temppred;
    merge subsub out3;
    run;

    /* keep only most recent contact attempt*/
    proc sort data=temppred; by caseid descending callday; run;
    proc sort data=temppred nodupkey out= daily_b&i. (keep= caseid callday runday daily_rp_est

    /* append the most up-to-date response propensity for each case on each day */
    %if &i. = &s. %then %do;
        data daily_bayes_phat;
        set daily_b&i.
        run;
    %end;
    %if &i. ne &s. %then %do;
        data daily_bayes_phat;
        set daily_bayes_phat daily_b&i.;
        run;
    %end;
%end;
/* order daily predictions */
proc sort data=daily_bayes_phat; by caseid runday; run;

/* append final reponse propensity to daily estimate for each case */
proc sql;
create table rp_est.bayes_phats as
select a.*, "Bayes" as method, b.final_phat
from daily_bayes_phat as a
left join final_phat as b
on a.caseid = b.caseid
order by a.caseid, a.callday
;quit;
```

```
%mend;
%byday(7,84);


/* append files from both methods */
/* files should each have caseid method callday runday daily_rp_est and final_phat*/
data rp_est.phat_eval;
set rp_est.current_phats rp_est.bayes_phats;
run;

/* look at difference between today's predicted probability and the final predicted probability */
data rp_est.phat_eval;
set rp_est.phat_eval;
    phat_diff = daily_rp_est - final_phat;
run;
/* compute mean and standard error of differences */
proc summary data=rp_est.phat_eval;
by method runday;
var phat_diff;
output out=diffdata mean=mean_dailydiff stderr=stderr_dailydiff;
run;
```
